# Tau pathology reduction with SM07883, a novel, potent, and selective oral DYRK1A inhibitor: A potential therapeutic for Alzheimer's disease

**DOI:** 10.1111/acel.13000

**Published:** 2019-07-03

**Authors:** Benoît Melchior, Gopi Kumar Mittapalli, Carolyn Lai, Karen Duong‐Polk, Joshua Stewart, Bora Güner, Brian Hofilena, Amanda Tjitro, Scott D. Anderson, David S. Herman, Luis Dellamary, Christopher J. Swearingen, K.C. Sunil, Yusuf Yazici

**Affiliations:** ^1^ Samumed, LLC San Diego California USA

**Keywords:** Alzheimer's disease, DYRK1A, mouse models, neurodegenerative diseases, neuroinflammation, Tau

## Abstract

Dual‐specificity tyrosine phosphorylation‐regulated kinase‐1A (DYRK1A) is known to phosphorylate the microtubule‐associated tau protein. Overexpression is correlated with tau hyperphosphorylation and neurofibrillary tangle (NFT) formation in Alzheimer's disease (AD). This study assessed the potential of SM07883, an oral DYRK1A inhibitor, to inhibit tau hyperphosphorylation, aggregation, NFT formation, and associated phenotypes in mouse models. Exploratory neuroinflammatory effects were also studied.

SM07883 specificity was tested in a kinase panel screen and showed potent inhibition of DYRK1A (IC_50_ = 1.6 nM) and GSK‐3β (IC_50_ = 10.8 nM) kinase activity. Tau phosphorylation measured in cell‐based assays showed a reduction in phosphorylation of multiple tau epitopes, especially the threonine 212 site (EC_50_ = 16 nM). SM07883 showed good oral bioavailability in multiple species and demonstrated a dose‐dependent reduction of transient hypothermia‐induced phosphorylated tau in the brains of wild‐type mice compared to vehicle (47%, *p* < 0.001). Long‐term efficacy assessed in aged JNPL3 mice overexpressing the P301L human tau mutation (3 mg/kg, QD, for 3 months) exhibited significant reductions in tau hyperphosphorylation, oligomeric and aggregated tau, and tau‐positive inclusions compared to vehicle in brainstem and spinal cord samples. Reduced gliosis compared to vehicle was further confirmed by ELISA. SM07883 was well tolerated with improved general health, weight gain, and functional improvement in a wire‐hang test compared to vehicle‐treated mice (*p* = 0.048).

SM07883, a potent, orally bioavailable, brain‐penetrant DYRK1A inhibitor, significantly reduced effects of pathological tau overexpression and neuroinflammation, while functional endpoints were improved compared to vehicle in animal models. This small molecule has potential as a treatment for AD.

## 1 INTRODUCTION

There remains an urgent need for treatments to improve or slow progressive neurodegenerative diseases (Cummings, Lee, Ritter, & Zhong, 2018). Tau pathology is considered a key driver for a broad spectrum of neurodegenerative diseases, collectively known as tauopathies (Iqbal, Liu, & Gong, 2015). These include primary tau diseases such as frontotemporal lobar degeneration with tau inclusions (FTLD‐tau), progressive supranuclear palsy (PSP), and Pick's disease, as well as multimodal diseases such as Alzheimer's disease (AD). In AD, tau aggregation and spreading appears to be enabled by amyloid‐beta aggregation, but therapeutics aimed at regulating the amyloid cascade have failed to prevent disease progression in symptomatic patients (Giacobini & Gold, 2013; Holtzman et al., 2016). In the context of a rapidly growing AD population with no disease‐modifying therapeutics, targeting tau appears to be a plausible approach; the spatiotemporal pattern of tau pathology in AD is highly correlated with brain atrophy and observed cognitive decline (Giannopoulos, Chiu, & Praticò, 2018). Tau is a multifunctional protein, which may become neurotoxic as a result of post‐translational modifications including a high degree of phosphorylation that leads to its oligomerization, sequestration, and aggregation (Spillantini & Goedert, 2013). Tau can be excessively phosphorylated at multiple epitopes, and these toxic forms of hyperphosphorylated tau (pTau) are found in higher quantities in AD brains. The buildup of insoluble oligomeric forms and gradual deposition of filamentous aggregated tau protein into intraneuronal pretangles and neurofibrillary tangles (NFTs) characterize all tauopathies (Bodea, Eckert, Ittner, Piguet, & Götz, 2016; Morris, Maeda, Vossel, & Mucke, 2011). While a pleiotropy of therapeutics has recently been tested in clinical trials to prevent tau aggregation and spreading, the exact form(s) of tau responsible for its toxicity and spreading remain to be identified (Khanna, Kovalevich, Lee, Trojanowski, & Brunden, 2016). Therefore, regulating the tau cascade upstream at the phosphorylation level is an appealing strategy.

Over 80 epitopes can be phosphorylated on tau, and more than 40 have been identified to be phosphorylated specifically in AD brains, with each pTau site having some degree of specificity for various kinases (Martin et al., 2013; Polanco et al., 2017; Šimić et al., 2016). Drug development has targeted inhibition of downstream kinases such as the mitogen‐activated protein kinase (MAPK) family or glycogen synthase kinase‐3 beta (GSK‐3β); both have shown benefit in preclinical models, however, thus far, without successful clinical outcomes (Le Corre et al., 2006; Lovestone et al., 2015; Onishi et al., 2011; Vogel et al., 2009). While multiple kinase inhibitors have recently been approved by the FDA, primarily in oncology, none have been approved for CNS disorders (Wu, Nielsen, & Clausen, 2015). Targeting kinases remains a viable approach to reduce tau phosphorylation and requires careful development to ensure brain penetrance and therapeutic efficacy.

The dual‐specificity tyrosine phosphorylation‐regulated kinase‐1A (DYRK1A) is a novel protein kinase that can directly and indirectly regulate phosphorylation of tau on numerous residues (Azorsa et al., 2010; Ryoo et al., 2007; Song et al., 2015; Walte et al., 2013). Overproduction of DYRK1A has been linked to increased tau phosphorylation and aggregation and is a likely contributor to higher incidence of AD in the Down syndrome (DS) population (Liu et al., 2008; Wegiel, Gong, & Hwang, 2011). Due to its localization on chromosome 21, the DYRK1A gene is 1.5‐fold overexpressed in DS and increased activity of DYRK1A has been correlated with abnormal brain development, cognitive disabilities, and an early onset of AD in individuals with DS (Becker, Soppa, & Tejedor, 2014). Furthermore, DYRK1A‐induced tau phosphorylation has been shown to disrupt the normal function of tau as a microtubule stabilizer and promote its aggregation (Liu et al., 2008). Mice overexpressing DYRK1A have increased pTau in the brain, while crossing DYRK1A heterozygous mice with DS mice regulated pTau, amyloid load, and symptoms (García‐Cerro, Rueda, Vidal, Lantigua, & Martínez‐Cué, 2017), implying DYRK1A is a highly dosage‐sensitive gene. Finally, in AD patients, overexpression of DYRK1A was observed in postmortem brains, further suggesting its contribution to tau hyperphosphorylation and the accumulation of NFTs (Ferrer et al., 2005). Targeting DYRK1A appears to be a viable treatment approach for AD. It is hypothesized that inhibition of DYRK1A activity may reduce tau pathology and inflammation and thus prevent, slow, or reverse AD or other chronic tauopathies.

SM07883 was developed by rational design as an orally available, brain‐penetrant, small‐molecule DYRK1A kinase inhibitor and showed regulation of multiple phospho‐tau epitopes both in vitro and in vivo in mouse brains. Repeat dosing in transgenic mice expressing brainstem tau pathology demonstrated efficacy in reducing formation of insoluble tau fragments and the subsequent cascade leading to tau aggregation, formation of tau‐positive inclusions, and associated neuroinflammation. Ultimately, loss of function and morbidity were found to be limited with SM07883 treatment compared to controls.

## 2 RESULTS

### 2.1 Discovery and pharmacological properties of SM07883, a potent DYRK1A kinase inhibitor

**Figure 1:**
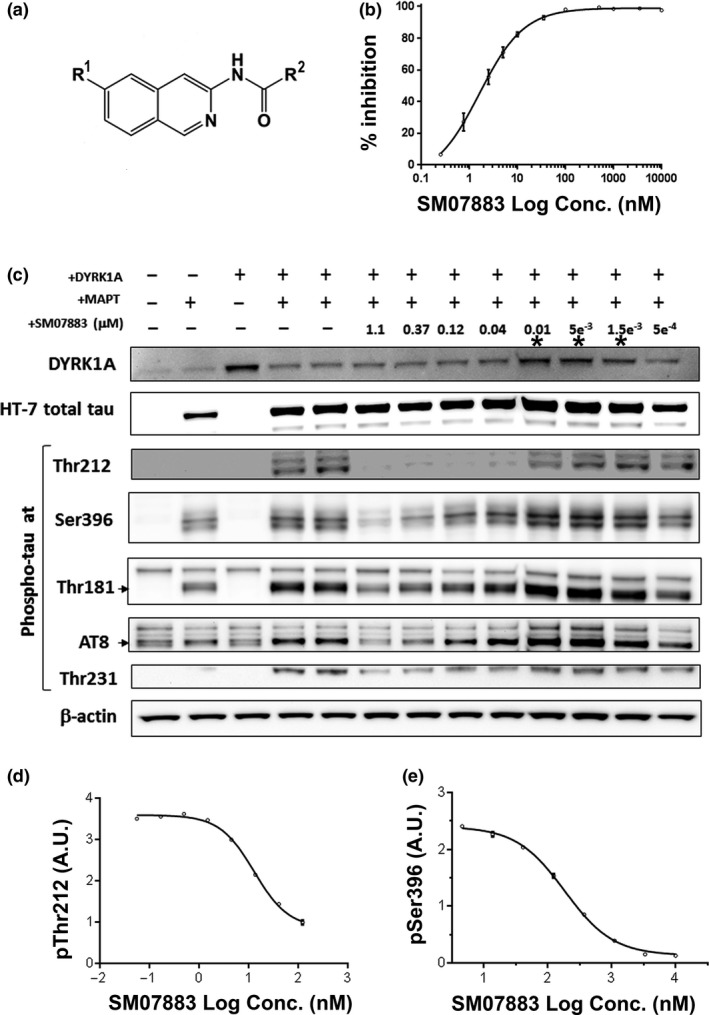
Discovery of SM07883 as a potent and selective DYRK1A inhibitor and regulator of tau phosphorylation. (a) Markush structure of SM07883, a 3‐acylamino‐isoquinoline analog, where R^1^ is (un)substituted 5‐membered heteroaryl, and R^2^ is selected from either (un)substituted C1‐4 alkylene‐heterocyclyl, or (un)substituted heterocyclyl, or (un)substituted C1‐4 alkylene‐carbocyclyl, or (un)substituted carbocyclyl as referred in WO, A2. (b) SM07883 inhibition of DYRK1A kinase activity using the Z‐Lyte™ platform. An IC_50_ value of 1.6 nM was determined from this 11‐point dose–response curve. (c) Western blot of HEK293T (co‐transfected with human DYRK1A and MAPT genes) cell lysates collected after 16‐hr treatment. SM07883 dose‐dependently inhibited potentiated tau phosphorylation. EC_50_ values with ratios over β‐actin are provided in Figure S1b. Asterisks indicate samples showing higher loading artifact. (d) An example of EC_50_ of 13 nM generated for this lot of SM07883 using an ELISA format elaborated for screening purposes of phosphorylated tau at Thr212. (e) A secondary screen was performed in SH‐S5Y5 human neuroblastoma cells. A dose‐dependent reduction of phosphorylated tau at Ser396 generated an EC_50_ of 184 nM for this lot of SM07883. Note: All IC_50_/EC_50_ curves are shown as mean of duplicates ± *SEM* (some *SEM* bars smaller than data point symbol)

SM07883 is a small‐molecule 3‐acylamino‐isoquinoline analog (Figure 1a), as referred in PCT patent application publication number WO, 2017189823, that was rationally designed and optimized through iterative medicinal chemistry to achieve significant exposure of the drug in the brain with oral administration. Physicochemical and ADME properties are provided in Table S1. SM07883 was designed as an ATP‐competitive kinase inhibitor with intrinsic and reversible blocking activity. SM07883 strongly inhibited DYRK1A activity in a dose‐dependent manner with a 50% inhibitory concentration (IC_50_) of 1.6 nM (Figure 1b). SM07883 was highly selective for DYRK1A on a panel of 416 kinases with only five family‐related kinase IC_50_ values within a 15‐fold range of DYRK1A inhibition (Table 1 and Figure S1): CLK4: 3 nM; DYRK1B: 8 nM; GSK‐3α and GSK‐3β: 11 nM; and DYRK2: 16 nM.

**Table 1:** IC_50_ of kinases inhibited by SM07883

	Full IC_50_ (nM)	Fold over DYRK1a
DYRK1a	1.6	–
CLK4	2.8	2
DYRK1b	7.9	5
GSK‐3β	11	7
GSK‐3α	11	7
DYRK2	16	10
CLK1	29	18
CDK9/cyclin T1	30	19
CDKL5	34	21
CDK16 (PCTK1)/cyclin Y	56	35
MYLK2 (skMLCK)	57	36
STK17A (DRAK1)	76	48
DYRK3	78	49
MAPK15 (ERK7)	90	56
CDK2/cyclin A	94	59
CLK2	99	62
TAOK1	101	63
MLCK (MLCK2)	109	68
CDK9/cyclin K	144	90
LRRK2	152	95
CDK5/p25	240	150
MAP4K4 (HGK)	496	310
MINK1	861	538
CDK1/cyclin B	1,018	636

Note

A whole‐panel screen of 416 native kinases was performed at a single dose of 1 μM of SM07883 and IC_50_ values from 11‐point titration curves were determined for the top 24 hits.

The ability of SM07883 to reduce tau phosphorylation was evaluated in 2 cell‐based assays utilizing double transfection and overexpression of both human microtubule‐associated protein tau (MAPT) and DYRK1A genes in HEK293T cells followed by Western blotting and confirmation by ELISA. In HEK293T cells, the dual transfection allowed for higher expression and detection of tau phosphorylation at multiple sites with pronounced effect at Thr212, Ser396, and Thr181 (Figure 1c). Dual transfection also potentiated the phosphorylation signal with AT8 (an antibody which binds to the pSer202/Thr205 sites) and the Thr231 epitope. After SM07883 treatment, a dose‐dependent decrease in phosphorylation was observed for all the tested epitopes with a calculated EC_50_ ranging from 7 nM for Thr212 to 228 nM for Ser396 (Figure S1b). When evaluated with ELISA allowing multiple screens over time, SM07883 was shown to inhibit phosphorylation at Thr212 with an average EC_50_ of 16 nM (±9 s.e.m.; Figure 1d) across tested lots.

SM07883 was tested in unstimulated, fast‐dividing SH‐SY5Y neuroblastoma cells, in which a high level of phosphorylated tau at Ser396 was shown to be reduced by overnight (16 hr) treatment when compared to vehicle (EC_50_ of 200 nM; ±66 s.e.m. across tested lots; Figure 1e). Screening of published DYRK1A and GSK‐3β inhibitors showed that tau phosphorylation at Ser396 was preferentially regulated by GSK‐3β inhibitors, while DYRK1A inhibitors had higher inhibitory activity at the Thr212 site (Table S2). SM07883 was the only compound screened with the ability to inhibit tau phosphorylation at both the Thr212 and Ser396 epitopes with nanomolar potency.

Although the most common post‐translational modification of tau proteins is phosphorylation, *O*‐glycosylation and acetylation may also affect localization, degradation, and function and are closely associated with the level of phosphorylated tau (Carlomagno et al., 2017; Liu et al., 2002). Regulation of acetylated and glycosylated tau was investigated in unstimulated SH‐SY5Y cells exposed to overnight treatment with SM07883. Western blot analysis showed only a marginal reduction at the highest tested dose when staining for glycosylated tau at serine 400 with N‐acetyl‐d‐glucosamine (*O*‐GlcNAc; Figure S2). A modest decrease in tau acetylation at K280 was observed but remained minimal compared to the reduction in tau phosphorylation at Ser396 observed at all tested doses.

Inhibition of CLK4 has been suggested to have a role on inhibition of SR protein phosphorylation with consequences on regulation of splicing and inclusion of tau exon 10 (Hartmann et al., 2001). Since SM07883 inhibited CLK4 in vitro, the compound was tested overnight on SH‐SY5Y cells and RNA was collected. The compound showed no regulation of the 3R/4R ratio of the MAPT gene encoding for tau using specific primers on flanking regions of exon 10 compared to control using RT–PCR. However, the use of a potent CLK2 inhibitor (Cmpd #79, USPTO patent application publication number US20160008365A1; CLK2 IC_50_: 9 nM) showed an evident reduction in the 4R form of tau indicative of splicing of exon 10 (Figure S3). Furthermore, no inhibition of SR phosphorylation was observed by Western blot analysis after 1‐hr incubation of SM07883 on SH‐SY5Y cells while the CLK2 inhibitor Cmpd #79 dramatically reduced phosphorylation of SRSF4 and SRSF6 at all tested doses (Figure S4).

### 2.2 Pharmacodynamic studies in wild‐type mice

**Figure 2:**
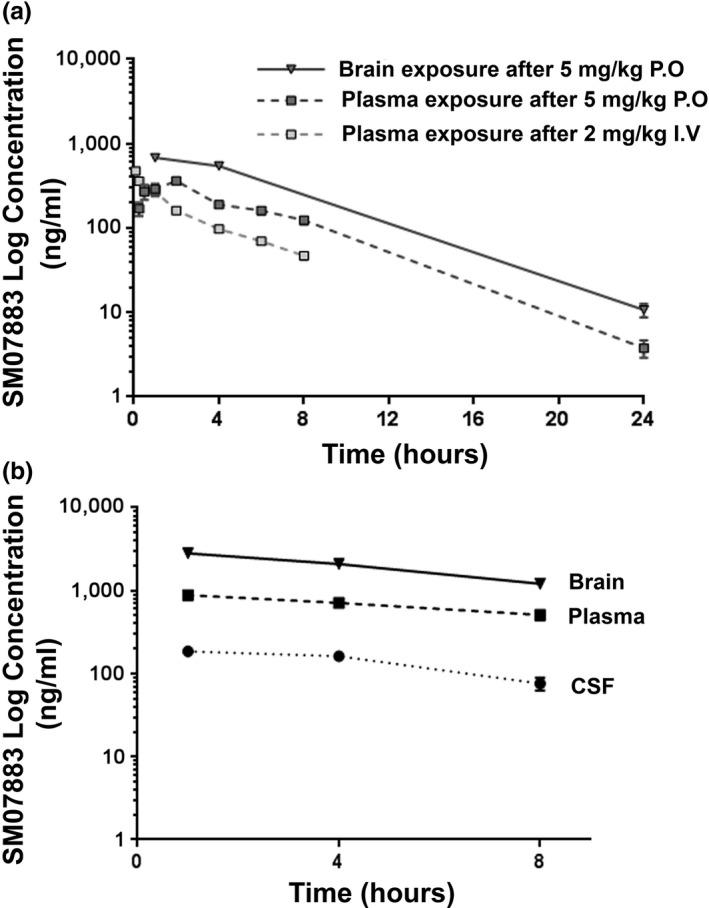
SM07883 is orally bioavailable and brain‐penetrant. (a) Single dose of SM07883 administered to BALB/c mice (P.O. 5 mg/kg of body weight, ■ or I.V. 2 mg/kg of body weight, □). Plasma concentration was quantified by mass spectrometry (*n* = 3 per time point). Brain tissues were also collected from the oral arm at 1 hr, 4 hr, and 24 hr (▼, *n* = 3 per time point). (b) Apparent log‐linear correlation in pharmacokinetic parameters in the brain (▼), plasma (■), and CSF (collected from the cisterna magna [●]) after an oral administration of 10 mg/kg of SM07883 in BALB/c mice (*n* = 3 per time point)

Following oral administration in mice, SM07883 was well absorbed with peak plasma concentration (Cmax) occurring 2 hr postdose and with an estimated bioavailability of 92% (Figure 2a). Pharmacodynamic properties were consistent across rodents and higher species (Table S3). The estimated terminal half‐life of 3.3 hr was suitable for once‐daily administration in mice. Additionally, low efflux was determined in vitro in Caco‐2 or MDR1‐MDCK cells (Table S1) and significant brain penetration was observed with a brain‐to‐plasma (Kp) ratio of 1.9 in mice. SM07883 showed approximately dose‐log‐linear increases in exposures (plasma and brain) across the pharmacodynamics (single dose) and tau transgenic mouse (3‐month daily doses) models. In vitro free fraction in rodent brain homogenate was 6% and correlated well with the in vivo distribution of SM07883 in plasma, brain, and CSF (Figure 2b). An additional study in rats showed SM07883 to be homogenously distributed throughout brain regions (Figure S5).

#### 2.2.1 SM07883 reduced tau phosphorylation in vivo

**Figure 3:**
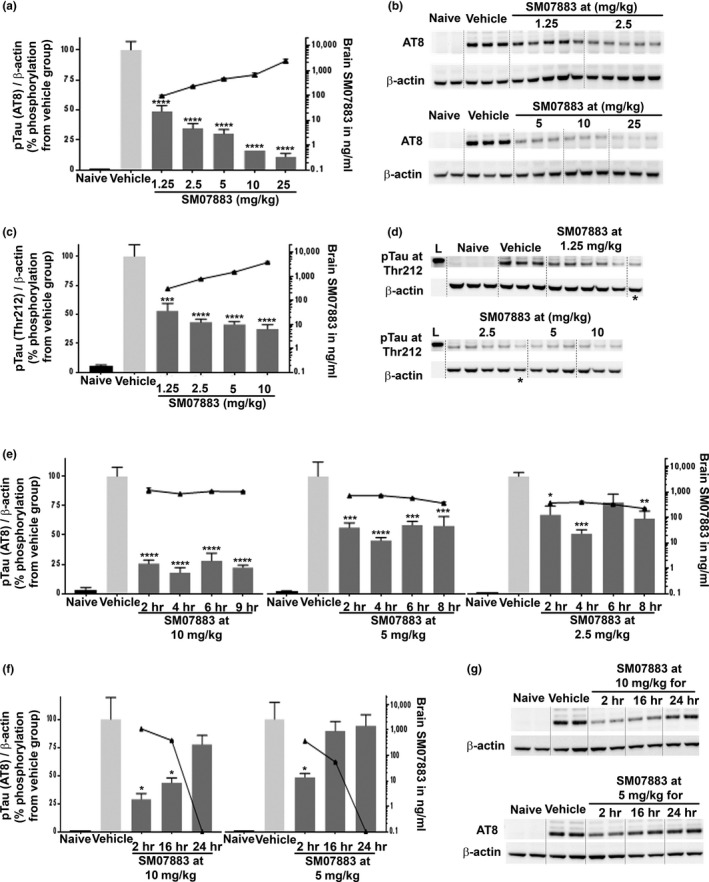
SM07883 reduced hypothermia‐induced tau phosphorylation in a dose‐dependent manner and correlated with increasing SM07883 brain exposure. BALB/c mice were administered a single oral dose of vehicle or SM07883 before hypothermia‐induced anesthesia before brain collection and analysis by Western blotting for tau phosphorylation at AT8 (Ser202/Thr205) or Thr212; β‐actin and ratio are displayed as bars (left *y*‐axis), while SM07883 exposure is shown as lines (right *y*‐axis). (a) Dose–response after 1 hr of anesthesia treatment (4 hr postvehicle (*n* = 3)) or SM07883 administration (*n* = 3–5). (b) Western blots used for densitometry results of AT8 (top) and β‐actin (bottom) for each tested dose. (c–d) Dose–response with blotting for tau phosphorylation at Thr212 (*n* = 3–5). Asterisks represent a loading control sample loaded on both gels, L is the ladder mark showing the 60 kDa size band. (e) Time course of brain collection from 2 to 8 hr postadministration of 10 (left), 5 (middle), or 2.5 mg/kg (right) of SM07883, while hypothermia induction remained induced 1 hr prior to sacrifice [corresponding Western blots are shown in Figure S6b]. (f–g) Extended time course (10 mg/kg, left; and 5 mg/kg right) with hypothermia 1 hr prior to sacrifice showing clearance of SM07883 at 24 hr and reduction of the tau signal. *p < 0.05, **p < 0.01, ***p < 0.001, ****p < 0.0001, one‐way ANOVA

Inhibition of tau hyperphosphorylation (pTau) in vivo by SM07883 was tested in an anesthesia‐induced transient tau hyperphosphorylation mouse hypothermia model (Bretteville et al., 2012). Phosphorylation of tau at Ser202/Thr205 sites was significantly increased by anesthesia in vehicle‐treated versus untreated control brain lysates when analyzed by AT8 Western blotting (Figure 3a,b, black vs. light gray bar, *p* < 0.0001). In this model, a single dose of SM07883 reduced hyperphosphorylated tau compared to the vehicle‐treated animals (Figure 3a, dark gray bars vs. light gray, *p* < 0.005) with the lowest dose of 1.25 mg/kg reducing tau phosphorylation by 47% (AT8/β‐actin ratio). Anesthesia‐induced tau phosphorylation declined in a dose‐dependent manner with increased SM07883 brain exposure (Figure 3a, black line). Phosphorylation of the Thr212 epitope on tau was also significantly affected in this model and followed the same inverted correlation with SM07883 brain exposure (Figure 3c,d, *p* < 0.005). SM07883 had no effect on regulating anesthesia‐induced decrease in body temperature (Figure S6a), suggesting these effects were due to direct regulation of tau phosphorylation. A single oral dose of 2.5, 5, or 10 mg/kg of SM07883 showed that inhibition of the signal was the strongest at 4 hr after administration and the reduction in tau phosphorylation remained significant at 8 hr (Figure 3e, *p* < 0.005). Longer time‐course studies indicated that the inhibitory signal persisted overnight (16 hr) in the 10 mg/kg group, although not significantly. However, only a marginal reduction in tau phosphorylation remained at 24 hr and correlated with no detectable amounts of SM07883 in the brain (Figure 3f,g).

### 2.3 SM07883 reduces tau pathology and motor task deficits in JNPL3 tau transgenic mice

#### 2.3.1 Reduction of pathological tau in SM07883‐treated brains

**Figure 4:**
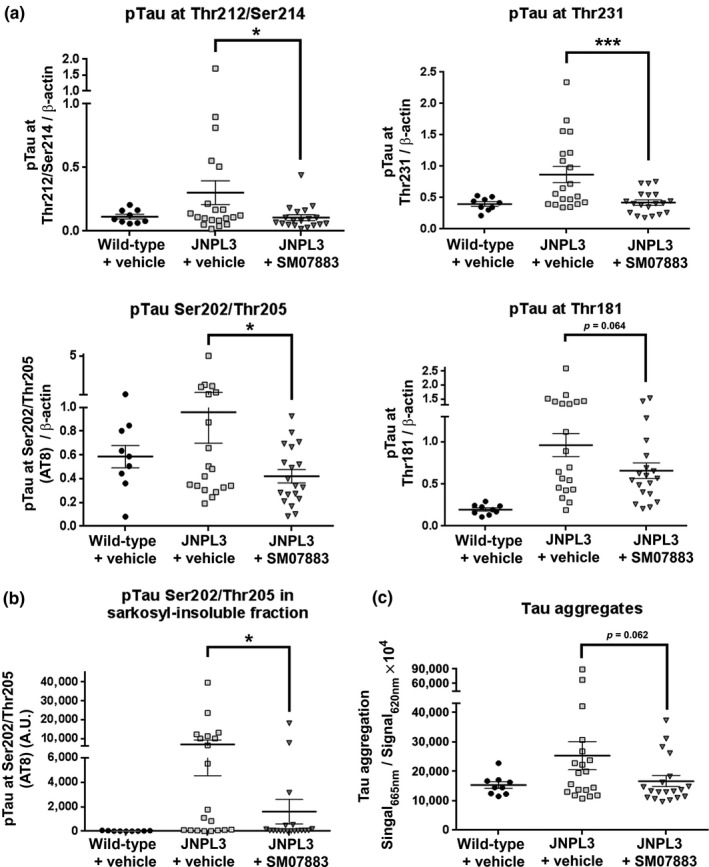
Reduction of pathological tau signals with treatment by SM07883. Brainstems and spinal cords from JNPL3 mice were collected after 3 months of a once‐daily dose of vehicle (*n* = 20, □), SM07883 (3 mg/kg, *n* = 19, ▼), or wild‐type littermates administered the vehicle only (*n* = 9, ●) with *p < 0.05 and ***p < 0.001 when compared to vehicle. (a) SM07883 significantly reduced pTau in the brainstem compared to JNPL3 mice treated with the vehicle. Lysates that were analyzed by Western blot for tau phosphorylation at Thr212/214, Thr231, AT8 sites (Thr202/Ser205), Thr181, and ratio over β‐actin were determined by densitometry. The AT8 monomer band of 55 kDa was used for this calculation. (b) SM07883 significantly reduced AT8 staining in the sarkosyl‐insoluble fraction from the brainstem compared to vehicle‐treated mice as analyzed by Western blotting (*p = 0.010). Only fragments ≥ 64 kDa were counted. Representatives blots can be found in Figure S7a (pTau) and S7B (sarkosyl). (c) SM07883 also demonstrated a reduction of specific forms of aggregated tau in spinal cord lysates (*p* = 0.062) from the same mice tested by HTRF assay with a pair of identical anti‐aggregated tau antibodies conjugated with two different fluorochromes

In a 3‐month, repeat‐dose efficacy study in JNPL3 tau mice, the brainstems and spinal cords were evaluated for tau pathology. The SM07883‐treated group (*n* = 19) showed a significant reduction in brainstem transgene‐induced phosphorylation of tau compared to JNPL3 mice treated with vehicle (*n* = 20; Figure 4a; Thr212/Ser214, *p* = 0.023; Thr231, *p* < 0.001; Ser202/Thr205 (AT8, 55 kDa band), *p* = 0.017; Thr181, *p* = 0.064). A similar trend was observed with a reduction of pTau at Ser396 (*p* = 0.223, Figure S8a) although the induction in JNPL3 mice compared to wild‐type mice was weaker for this epitope.

Similarly, SM07883 significantly reduced sarkosyl‐insoluble tau fragments visualized by AT8 staining in the brainstems compared to vehicle (Figure 4b; *p* = 0.010). The reduction in tau aggregates was also confirmed in the spinal cord lysates of the SM07883‐treated group compared with the vehicle group as measured by homogenous time‐resolved fluorescence (HTRF) (Figure 4c, *p* = 0.062), although the specific size of these fragments is unknown and their restricted pattern may likely have contributed to the low sample size.

**Figure 5:**
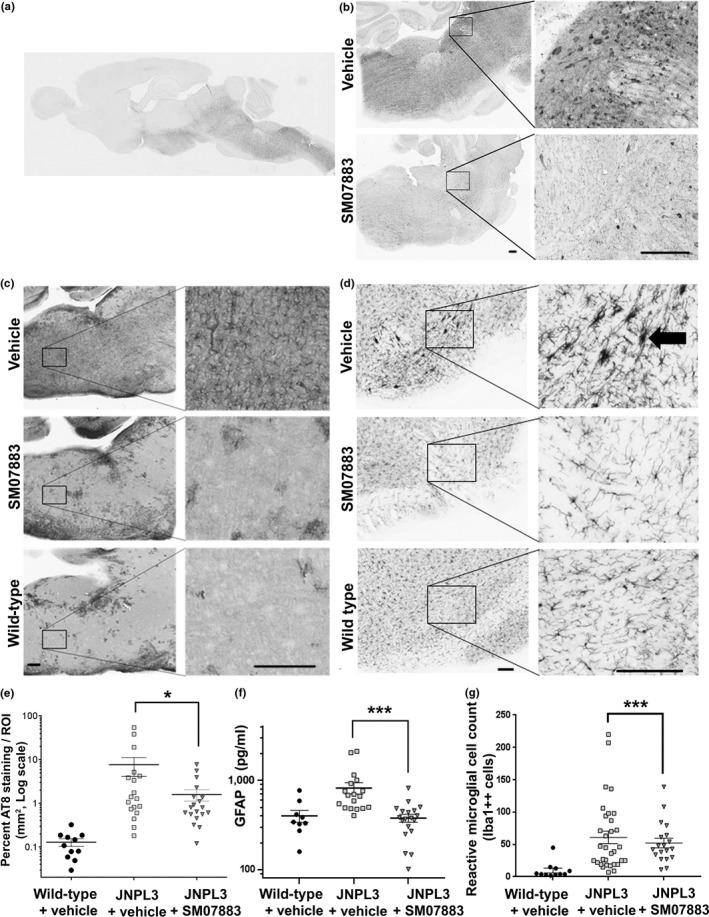
Reduction of tau‐positive inclusions and glial staining in JNPL3 mouse brains after treatment with SM07883. Hemispheres from JNPL3 mice were collected at the termination of 3 months of treatment of a once‐daily dose of vehicle (*n* = 20, □), SM07883 (3 mg/kg, *n* = 19, ▼), or wild‐type littermates administered the vehicle only (*n* = 9, ●), and sagittal sections were stained for AT8; scale bars are 200μm. (a) Global view of a vehicle‐treated JNPL3 mouse hemisphere stained with AT8 (4× magnification). (b) AT8 staining in a vehicle‐treated (top) or SM07883‐treated brainstem (bottom) with respective computer enlarged image on the right panels. (c) Micrographs showing GFAP staining of the brainstem from one JNPL3 treated with either vehicle (top); SM07883 (3 mg kg^−1^ day^−1^, middle); and wild‐type mouse brain treated with vehicle. Images were captured at 10× magnification. Left panel corresponds to an enlargement of the right image inset. (d) Micrographs showing Iba1 staining and dense cellular staining identified reactive microglia cells (arrow) in the same brainstems. (e) Quantification of tau‐positive inclusions was performed on the region of interest (ROI), which was delimited within the hindbrain area. The percent AT8 surface staining area over the ROI, to capture neurite extensions in addition to somatic staining, showed significantly higher staining in the vehicle group compared to SM07883‐treated mice (vehicle (*n* = 18): 7.7 ± 3.5; SM07883 (*n* = 19): 1.6 ± 0.5 (*p = 0.039)). (f) Protein lysates from JNPL3 and wild‐type spinal cord samples were analyzed by ELISA (In ng/ml, wild‐type: 399 ± 60; JNPL3 with vehicle: 820 ± 118, *n* = 18; SM07883: 378 ± 38, *n* = 19; JNPL3 vehicle versus SM07883, ***p < 0.001). (g) Quantification of bulky core microglial cells (Iba1++, arrow, D) shows a decrease in the number of reactive microglial cells in SM07883‐treated JNPL3 hindbrains (SM07883‐treated: 52 ± 1.5 *n* = 19 versus vehicle‐treated: 61 ± 2.9 *n* = 32; ***p < 0.001)

The other brain hemispheres were processed for AT8 immunostaining and revealed tau pathologies within the hindbrain of JNPL3 mice like those reported by Lewis et al. (2001; Figure 5a and Table S4). Increased magnification illustrated the presence of tau inclusions similar to intraneuronal tau tangles (Figure 5b) with a higher abundance in the vehicle group compared to SM07883‐treated group (Figure 5e; percent occupation by AT8 staining over the region of interest; *n* = 18 vehicle, *n* = 19 treated with SM07883, *p* < 0.039). The total amount of human transgenic tau was also found increased and is a likely consequence of increased accumulation of tau inclusions (Figure S8b). Compared to vehicle, significant reductions of pTau, aggregation of tau, and hindbrain AT8 staining were also observed with an alternative dose regimen of SM07883 (10 mg/kg daily or every other day; Figure S9).

#### 2.3.2 SM07883 reduced tau‐associated glial activation

Markers of neuroinflammation associated with tau pathology were also evaluated in JNPL3 brains and spinal cord samples. Representative micrographs in Figure 5c and Figure 5d show a reduction in gliosis (GFAP immunoreactivity) and the number of Iba1‐reactive microglial cells (*p* < 0.001, Figure 5g) following treatment with SM07883 compared with vehicle‐treated animals. Reduction in GFAP expression was quantified by ELISA in spinal cord samples from mice treated with SM07883 compared to both vehicle‐treated groups (*n* = 19 and *n* = 18, respectively; *p* < 0.001, Figure 5f).

#### 2.3.3 Improvement in weight loss, mortality, and morbidity in JNPL3 mice treated with SM07883

**Figure 6:**
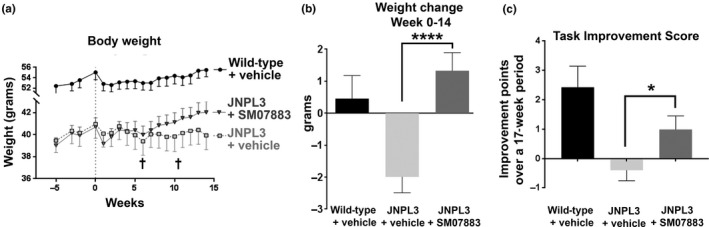
Improvement in weight loss, mortality, morbidity, and functional deficit in JNPL3 mice treated with SM07883. (a) Weekly body weight changes from 9 months of age until termination of the study at 13 months of age. JNPL3 mice were dosed once‐daily with either SM07883 (3 mg/kg, *n* = 19; ▼) or vehicle (*n* = 20; □) starting at 10 months of age; referred to week 0. Age‐matched wild‐type received the vehicle (*n* = 9). Cross signs indicate early deaths in the vehicle group. (b) Changes in body weight from start of dosing (week 0) to termination of the study (week 14) showed weight gain when JNPL3 mice were treated with SM07883, ****p = 0.0001 compared to vehicle (one‐way ANOVA). (c) SM07883‐treated mice demonstrated a positive improvement in the wire‐hang test over 17 weeks (3 weeks prior to administration to termination at 14 weeks) compared to the JNPL3 vehicle‐treated group (*p = 0.048, one‐way ANOVA)

Over the course of the 3‐month treatment, compared to vehicle, SM07883 showed a reduction in weight loss and mortality, which are inherent characteristics of the JNPL3 transgenic strain (Lewis et al., 2001). Initiation of daily dosing in all groups was accompanied by a noticeable drop in weight in all groups, most likely a consequence of the sudden daily manipulation of the mice (Figure 6a). While wild‐type mice recovered with weight gain by the end of the study, the vehicle‐treated JNPL3 mice showed a continued weight loss over the treatment time (Figure 6a, JNPL3 + vehicle: −2.0 g ± 0.5, *n* = 18, squares; and Figure 6b, light gray bar). Mortality and morbidity were also greater in the vehicle‐treated group. A greater number of moribund animals in this group contributed to the overall drop in weight, including two early deaths at 6 and 11 weeks (Figure 6a, †). The 2 early deaths were reflected in a rebound of the group's weight the following week. Vehicle‐treated mice showed signs of distress at the end of the study, with mild to severe tremors, a consequence of the expression of the transgene in the brainstem and spinal cord, and one individual with a pronounced hunched back posture (Table 2). Tremors were often associated with rough coat and reduced mobility. SM07883‐treated mice, however, showed a quick recovery in weight loss after initiation of the dosing (Figure 6a, triangles) and the group had a positive net gain over the course of the study (1.3 g ± 0.57, *n* = 19; Figure 6b, dark gray bar) as well as a reduction of the clinical signs (Table 2).

**Table 2:** Clinical observations at termination of the study in 13‐month‐old JNPL3 mice

Clinical stage	Symptoms	Wild type + Vehicle		JNPL3 + Vehicle			JNPL3 + SM07883		
		Frequency	%		Frequency	%		Frequency	%	
Presymptomatic	No symptom	9/9	100		8/18	44		17/19	89	
Early symptomatic	Mild tremors	0/9	0		6/18	33		2/19	11	
	Moderate hunched back	0/9	0		0/18	0		0/19	0	
Fully symptomatic	Severe tremors	0/9	0		3/18	17		0/19	0	
	Pronounced hunched back	0/9	0		1/18	6		0/19	0	
Early demise	Death	0/9	0		2/20	10		0/19	0	

Note

No clinical signs were observed in the wild‐type mice besides signs of age‐related obesity (not shown).

#### 2.3.4 Improvement of motor task performance with SM07883 treatment

As the pathological tau was mostly expressed in neurons of the hindbrain and spinal cord of aged JNPL3 mice (Figure 5a), these mice progressively displayed motor deficits related to brainstem pathology and were tested for motor coordination in the wire‐hang test. Over the course of the treatment, both the wild‐type and SM07883‐treated groups improved in task performance compared to vehicle‐treated groups with an increase of 2.4 ± 0.72 and 1.0 ± 0.46, and a decrease of 0.4 ± 0.36 points in the wild‐type, SM07883‐treated, and vehicle‐treated groups, respectively (Figure 6c; *p* = 0.048 between vehicle‐treated (*n* = 19) and SM07883‐treated (*n* = 19) groups).

## 3 DISCUSSION

There are no disease‐modifying therapies for AD, and despite continued attempts and dozens of clinical trials, the FDA has not approved any new treatment for AD in 15 years (Cummings et al., 2018). DYRK1A has been shown to regulate multiple pathways that contribute to tau pathology. Inhibition of DYRK1A may reduce these factors and the pathogenesis of AD. Proof‐of‐concept studies with DYRK1A inhibitors have demonstrated a reduction in amyloid pathology, insoluble tau phosphorylation, and neuroprotection in mice (Branca et al., 2017; Naert et al., 2015; Neumann et al., 2018). Previous attempts at modulating kinases in patients (GSK‐3β) may have failed due to the low potency and limited bioavailability of those compounds. Once‐daily oral SM07883, a potent DYRK1A inhibitor, significantly reduced the deleterious effects of pathological tau and associated neuroinflammation in a transgenic mouse model. SM07883 may regulate a novel target and provide potential therapeutic, disease‐modifying effects in AD.

SM07883 potently inhibited DYRK1A kinase activity with a novel selectivity profile. With single‐digit nanomolar potency and selectivity for DYRK1A, and only a handful of family‐related kinases, SM07883 presents a unique profile of inhibition of tau phosphorylation at multiple epitopes. This inhibition was found most pronounced at, but not limited to, the Thr212 site. Both in vitro and in the brain stem of JNPL3 mice, SM07883 reduced the phosphorylation all tested tau epitopes, all of which have been found highly phosphorylated in AD brains (Martin et al., 2015; Neddens et al., 2018; Oliveira, Costa, de Almeida, da Cruz e Silva, & Henriques, 2017; Šimić et al., 2016). This ability to prevent hyperphosphorylation at multiple sites may afford an advantage over single‐epitope‐targeted therapeutics in reducing tau neurotoxicity (Steinhilb, Dias‐Santagata, Fulga, Felch, & Feany, 2007). GSK‐3β inhibitors lacking DYRK1A inhibition tested in *vitro* in this study only demonstrated a restricted pattern of phosphorylation potentially due to the lack of Thr212 inhibition. Phosphorylation at this site also primes tau for further phosphorylation at additional sites by other kinases (Woods et al., 2001). Phosphorylation at Thr212 was further shown to be critical for the regulation of microtubule assembly (Ryoo et al., 2008; Wang, Grundke‐Iqbal, & Iqbal, 2007), the regulation of learning and memory (Beharry, Alaniz, & Alonso, 2013), and neurotoxicity (Steinhilb et al., 2007). Indeed, phosphorylated Thr212 is part of a combination of phosphorylation events at key sites, which precedes the formation of tau oligomers and causes memory impairments (Di et al., 2016). The Thr212 site was found to be hyperphosphorylated in patients with AD, which indicates that its regulation may contribute to disease pathogenesis (Morishima‐Kawashima et al., 1995; Ferrer et al., 2005; Yu et al., 2009). One unrelated kinase, CLK4, was regulated by SM07883, but in contrast to previously published studies (Hartmann et al., 2001), inhibition by SM07883 did not affect tau splicing or SR protein phosphorylation.

It is proposed that DYRK1A expression is regulated by a wide range of molecular changes and disease and exposure to stimuli such as the proinflammatory cytokine TNF‐α, cellular stress signals, or soluble beta‐amyloid (Aβ) was shown to induce DYRK1A expression, while in return, these increases in DYRK1A expression induced tau phosphorylation, Aβ production, and Aβ‐induced tau aggregation (Choi & Chung, 2011; Coutadeur et al., 2015; Kang, Choi, Park, & Chung, 2005). The higher levels of DYRK1A expression found in the brains of people with AD and Pick's disease and the overlap with hyperphosphorylated tau suggest that the kinase is contributing to tau disease pathogenesis (Ferrer et al., 2005). Treatment of JNPL3 mice with SM07883 significantly reduced pTau at multiple AD critical epitopes in the brainstem compared to vehicle; these results were like the effects seen in vitro. Consequently, SM07883 significantly reduced tau aggregation, leading to significantly lower numbers of tau‐positive inclusions. The reduction in brainstem pathology in JNPL3 mice treated with SM07883 was correlated with improved motor task performance, while vehicle‐treated mice worsened. The effects on weight, general well‐being, and survival rate indicated that SM07883 was well tolerated and prevented gross clinical signs of the disease compared to vehicle‐treated JNPL3 mice. There was associated reduction in neuroinflammation with a significant reduction in gliosis in the brainstem and spinal cord of JNPL3 mice treated with SM07883. It is not yet clear, in JNPL3 mice treated with SM07883, whether the reduction of neuroinflammation is due to a direct regulation of gliosis or an indirect consequence of the reduction of tau pathology. However, DYRK1A expression has been linked to regulation of inflammation and astrogliogenesis (Khor et al., 2015; Kurabayashi, Nguyen, & Sanada, 2015) and a direct effect of SM07883 in regulating glial activation could be expected.

Sustained systemic and brain exposure after oral administration demonstrated good bioavailability and blood–brain barrier penetrance, and made SM07883 suitable for once‐a‐day dosing in these studies. With high exposure in CNS compartments across multiple species and significant pharmacodynamics response in mice, these results indicate that reducing DYRK1A activity may be a viable treatment in diseases such as AD, where an increase in enzyme activity may regulate the cascade of phosphorylation leading to tauopathies and neurodegeneration.

## 4 EXPERIMENTAL PROCEDURES

### 4.1 Animals

Seven‐week‐old wild‐type (WT) male BALB/c mice (Envigo) were used for pharmacokinetics and pharmacodynamics studies. Sprague Dawley male rats (Charles River) were used for the compound brain distribution study. Six‐month‐old male transgenic Tg(PrnpMAPT*P301L) JNPL3Hlmc (“JNPL3”) and age‐matched wild‐type mice were purchased from Taconic Farms and allowed to age in‐house until they were 10 months of age. These mice were placed on a NIH #31M diet (Envigo) throughout the entire study.

### 4.2 Test article

For in vitro assays, 10 mM DMSO stock solutions of SM07883 were serially diluted in a 96‐well V‐bottom polypropylene plate and 8–11 concentration points with threefold dilution were tested. Final amounts of DMSO in overnight cell assays never exceeded 0.5% in media. For oral administration, SM07883 (1.25–25 for BALB/c and 3 mg/kg for JNPL3) or vehicle (5% PVP) was delivered at a constant volume of 150 µl per mouse.

### 4.3 Kinase assays

DYRK1A kinase, CLK2, and CDK1/cyclin B 50% inhibitory concentration (IC_50_) values were determined in a nonradioactive fluorescence response energy‐transfer antibody‐free biochemical assay using the Z‐Lyte™ platform with a serine/threonine 18 peptide substrate (Thermo Fisher Scientific (Thermo)). Eleven‐point dose–response curves were generated with experimental duplicates, and IC_50_ values were calculated using nonlinear regression curve. The selectivity was evaluated by the percent of inhibitory effect with a single point at 1 μM on a panel of 416 native kinases, and full IC_50_ curves were determined for top kinase hits defined by >80% inhibition at 1 μM and were performed by Thermo Fisher SelectScreen™ service.

### 4.4 Cells and cell‐based assays

Human embryonic kidney cells, HEK293T cells (ATCC), were cultured in Dulbecco's modified Eagle's medium [DMEM], 10% fetal bovine serum [FBS], and 1% penicillin/streptomycin), while human neuroblastoma SH‐SY5Y cells (Sigma) were cultured in 1:1 DMEM/F‐12 medium supplemented with 15% FBS and 1% nonessential amino acid and incubated at 37°C with 5% carbon dioxide. 1X neat radioimmunoprecipitation assay (RIPA, Cell Signaling Technology) buffer containing phosphatase and protease inhibitors was used to lyse cells. Transfection assay and ELISA are described in the supplementary materials.

### 4.5 Single‐dose pharmacodynamics in mice

Wild‐type BALB/c male mice (7 weeks old) were administered a single dose per oral gavage of vehicle or SM07883 (1.25, 2.5, 5, 10 or 25 mg/kg). Plasma and tissue processing for bioanalytical studies are described in the supplementary materials. For pharmacodynamics studies, one hour prior to brain and plasma collection mice were treated with a ketamine/xylazine (K/X) cocktail (150 µl of 100 mg/kg) via intraperitoneal (I.P.) administration to trigger anesthesia‐induced hypothermia and subsequent brain tau phosphorylation. Exactly one hour postinduction, mice were decapitated, and tissues were collected, flash‐frozen, and stored for Western blot analysis as described below. Untreated (nonanesthesia) control mice were sacrificed under CO_2_.

### 4.6 Repeat‐dose efficacy in tau transgenic mice and wire‐hang test

Nine wild‐type littermates and 39 tau JNPL3 male mice carrying the human P301L tau mutation form of tau from autosomal dominant tau FTD patients (Lewis et al., 2001) were randomized in groups after initial behavior testing (wire‐hang test). At 10 months of age, mice received once‐daily administration of SM07883 (3 mg kg^−1^ day^−1^, P.O.) or vehicle solution. The treatment was continuous for 96–98 days (~3 months). Motor coordination and strength were evaluated using the wire‐hang test, which measures agility and grip capacity. A composite of the falling score and a grip score were recorded as described in the supplementary materials. Results were reported from 3 trials/animal separated by 1 h. In addition to the weekly weight measurement, mice were regularly monitored for gross clinical score and clinical signs typical of the JNPL3 transgene‐induced disease phenotype (see supplementary materials). At the end of the treatment, animals were sacrificed with CO_2_ and brain and spinal tissue collected. Histology for intraneuronal tau pathology and neuroinflammation was performed after tissue was fixed in 10% neutral formalin, embedded, sectioned in the sagittal plan, and stained using biotinylated antibodies as detailed in the supplementary materials.

### 4.7 Western blotting and sarkosyl‐insoluble tau fragment detection

Cells, brainstem, or spinal cord samples were lysed in RIPA buffer containing protease and phosphatase inhibitors (Halt™, Catalog #78440, Thermo) and homogenized in CK14 tubes (Precellys, Bertin Instruments) at 5,000 rpm prior to centrifugation. Supernatants were mixed with Lithium Dodecyl Sulfate (LDS, Catalog #NP0007, Thermo) and reducing agent (Catalog #1610792, Bio‐Rad) prior to boiling. Samples were further diluted in 1X LDS and loaded onto a NuPAGE protein electrophoresis gel, then transferred onto nitrocellulose blots before blocking and blotting with a primary antibody followed by anti‐mouse HRP‐conjugated antibody and revealed by bioluminescence. Blots were probed for β‐actin for control of total protein loading. Primary antibodies used in this study can be found in Table S6. Western blot confirmation of the regulation of phosphorylated tau epitopes and other related DYRK1A/MAPT targets was analyzed through measurement of band densitometry using the NIH freeware ImageJ, and values were normalized as ratios over beta‐actin or total tau (HT7).

Homogenized brainstem samples in RIPA were further sonicated and separated by extraction in 1% sarkosyl solution for 1 h on an orbital shaker and ultracentrifugated at 150,000 *g* for 1 hr. After rinsing with RIPA and PBS, samples were air‐dried and sarkosyl‐insoluble pellets were resuspended in 1X LDS/10M urea and beta‐mercaptoethanol. Samples were further denatured at 95°C for 5 min prior to dilution, loading onto NuPAGE gels and blotting with an AT8 antibody as described above. Unless noted, no band at the 55 kDa size, expected to be monomeric tau, was detected and bands of 64 kDa and above were quantified.

To confirm the reduction of tau fragments, specific size of aggregated forms of tau were evaluated in spinal cord lysates by HTRF using a tau aggregation assay combining the same total tau antibody conjugate with a donor and acceptor fluorochrome (6FTAUPEG, Cisbio). According to the manufacturer, specific wavelength (665 nm) fluorescence is emitted if both antibodies are within 9 nM distance. Although the exact size range of tau aggregates detected in this assay is unknown, the assay does not recognize nonaggregated forms of tau. The plate was read with the Envision multiplate reader (PerkinElmer).

### 4.8 Statistical analysis

For dose–response, analysis of variance (ANOVA) with Dunnett's multiple comparison correction was used; the nonparametric Kruskal–Wallis test with Dunn's multiple comparison correction was also used as a sensitivity analysis (Figures 3 and 6).

Due to the positively skewed and strictly non‐negative response data, parametric generalized linear models assuming a gamma distribution (with reciprocal link function) were used to estimate differences between vehicle and treatment groups in the JNPL3 study (Figures 4 and 5). For severely skewed data where gamma distribution did not characterize the data well, rank‐based Wilcoxon–Mann–Whitney/Kruskal–Wallis tests were employed. A summary of statistical tests used in JNPL3 mice analysis is provided in Table S6.

## CONFLICT OF INTEREST

None declared.

## AUTHORS' CONTRIBUTION

BM, GM, SKC, and YY provided the concept, designed, and supervised the research; GM, CL, KDP, JS, BH, SDA, LD, BG, and AT carried out the experiments; BM, GM, CL, KDP, JS, DH, LD, CJS, SKC, and YY analyzed and interpreted the data; BM and DH drafted the manuscript. All authors read and approved the final manuscript.
