# Supplementary figures and images for: Tau pathology reduction with SM07883, a novel, potent, and selective oral DYRK1A inhibitor: A potential therapeutic for Alzheimer's disease
Source: Aging Cell. 2019 Jul 3;18(5):e13000. doi: 10.1111/acel.13000 (PMC6718548; doi:10.1111/acel.13000)

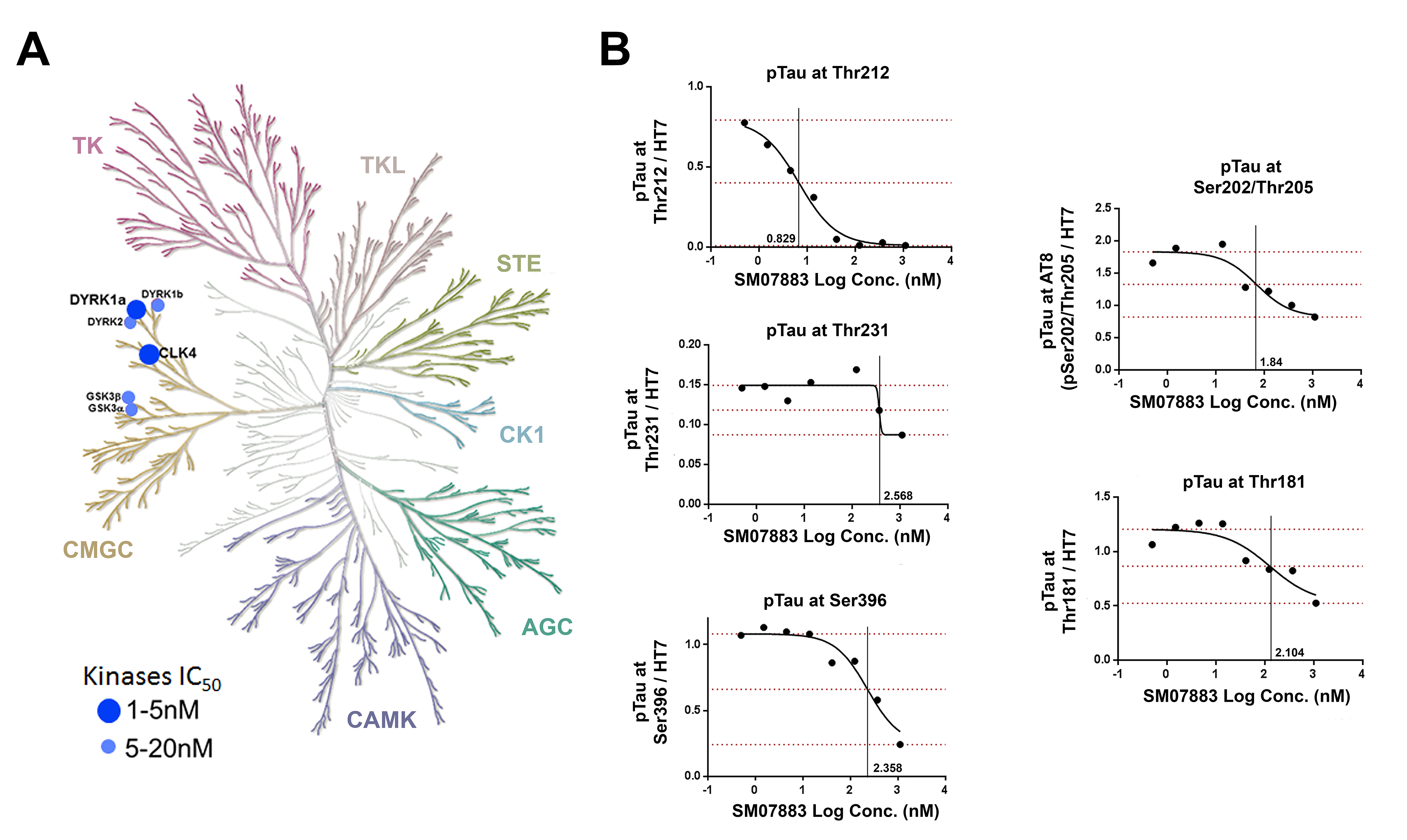

Supplement: Supplementary file 1 [file ACEL-18-e13000-s001.tif]

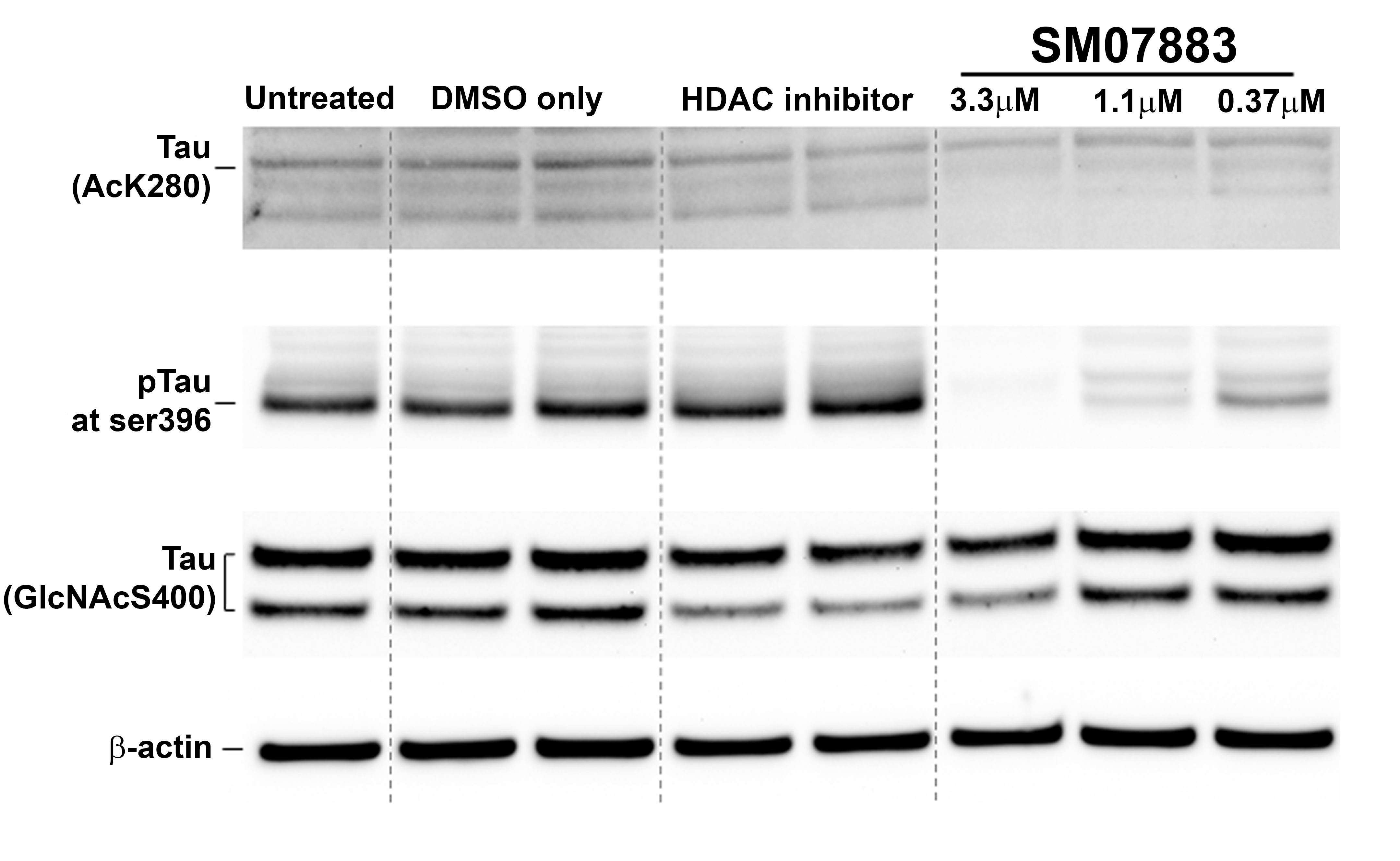

Supplement: Supplementary file 2 [file ACEL-18-e13000-s002.tif]

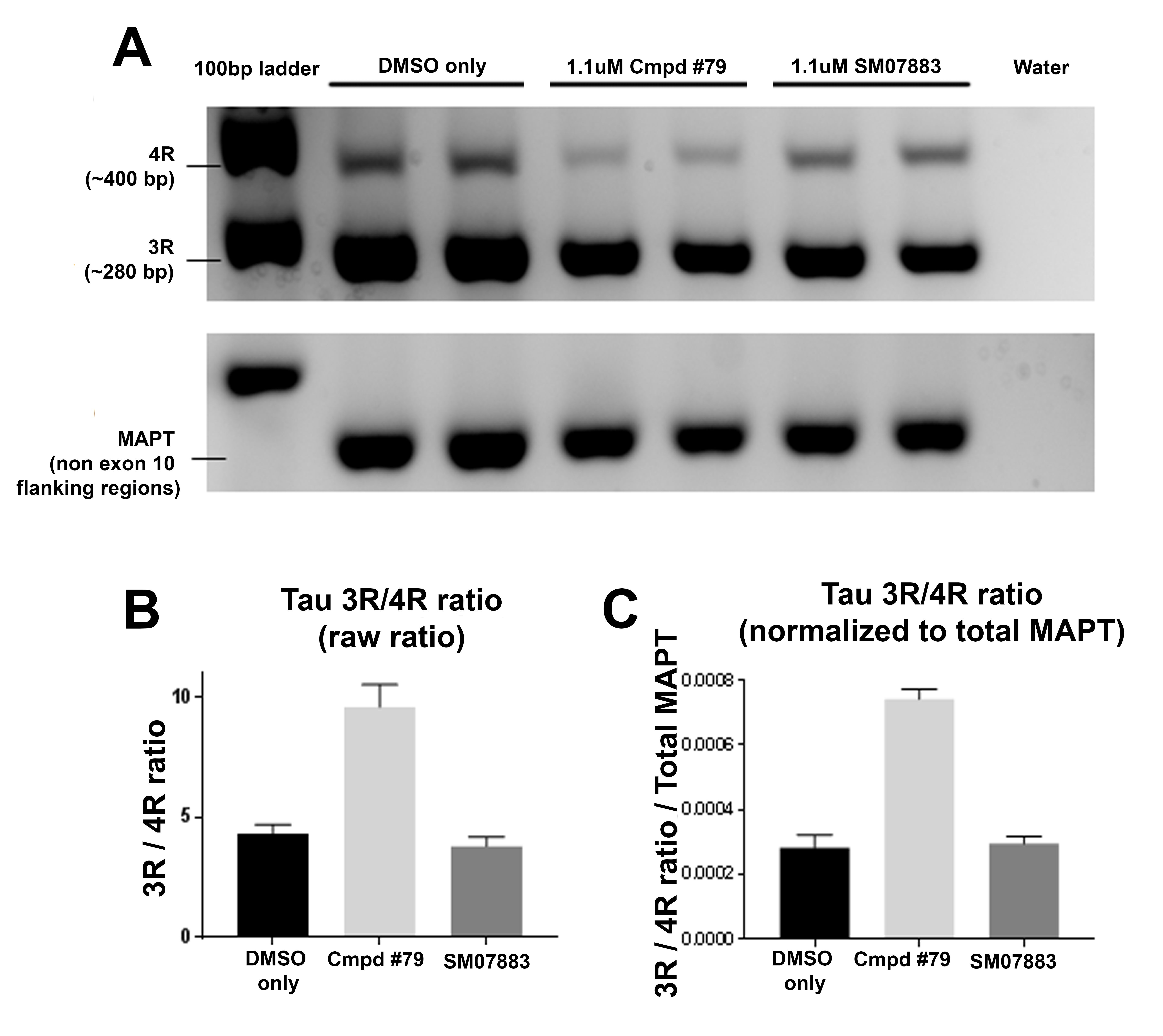

Supplement: Supplementary file 3 [file ACEL-18-e13000-s003.tif]

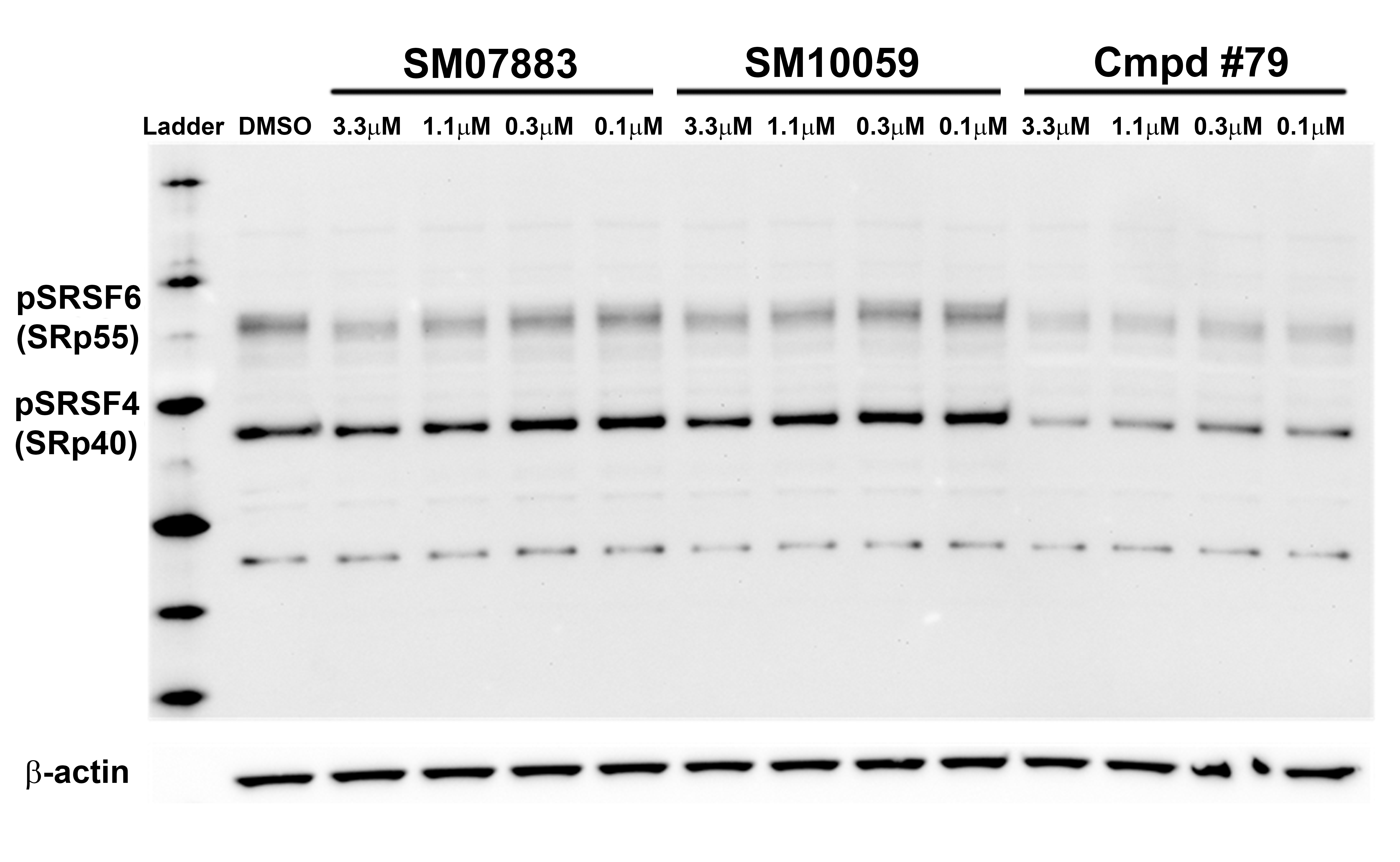

Supplement: Supplementary file 4 [file ACEL-18-e13000-s004.tif]

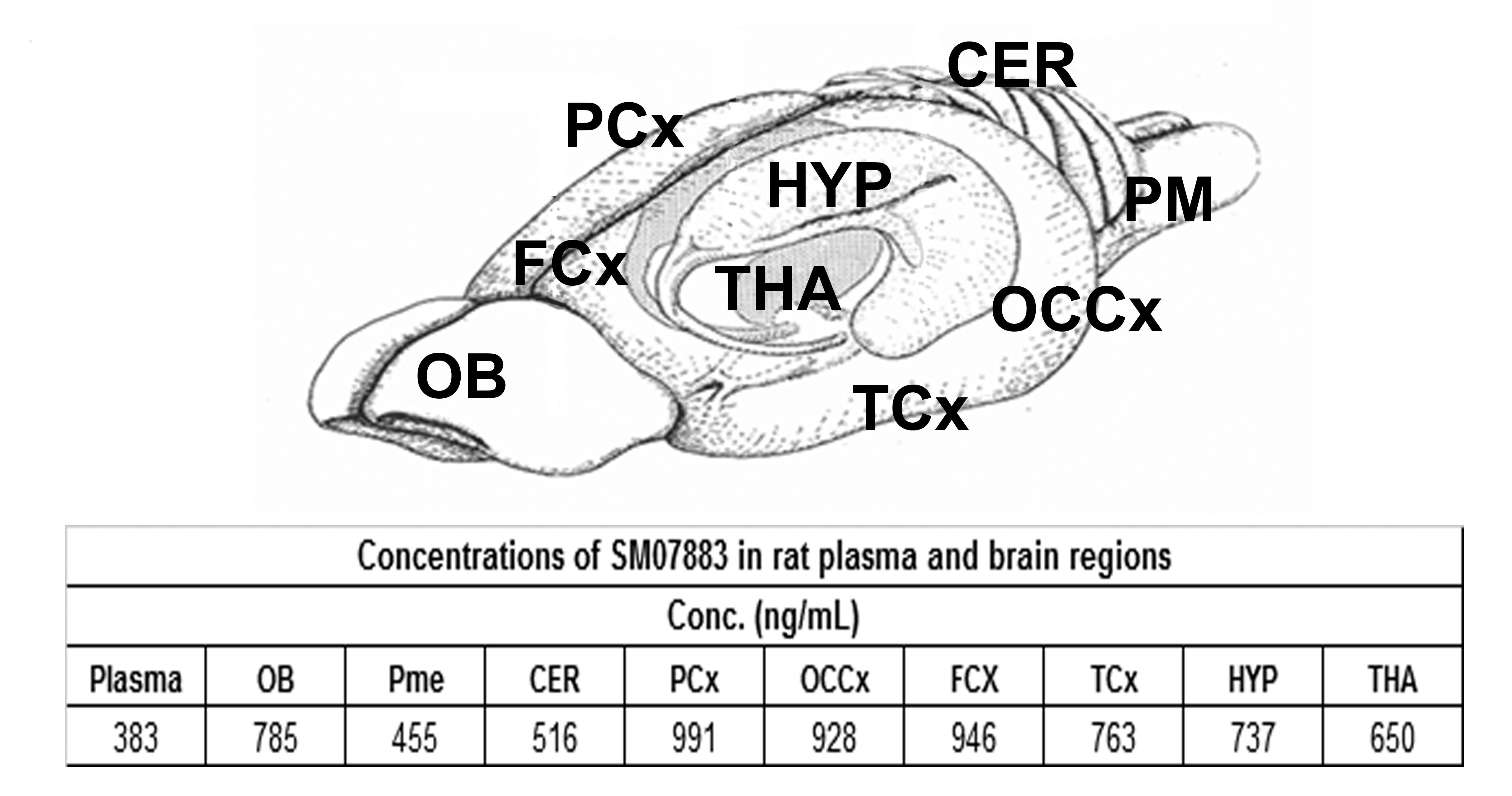

Supplement: Supplementary file 5 [file ACEL-18-e13000-s005.tif]

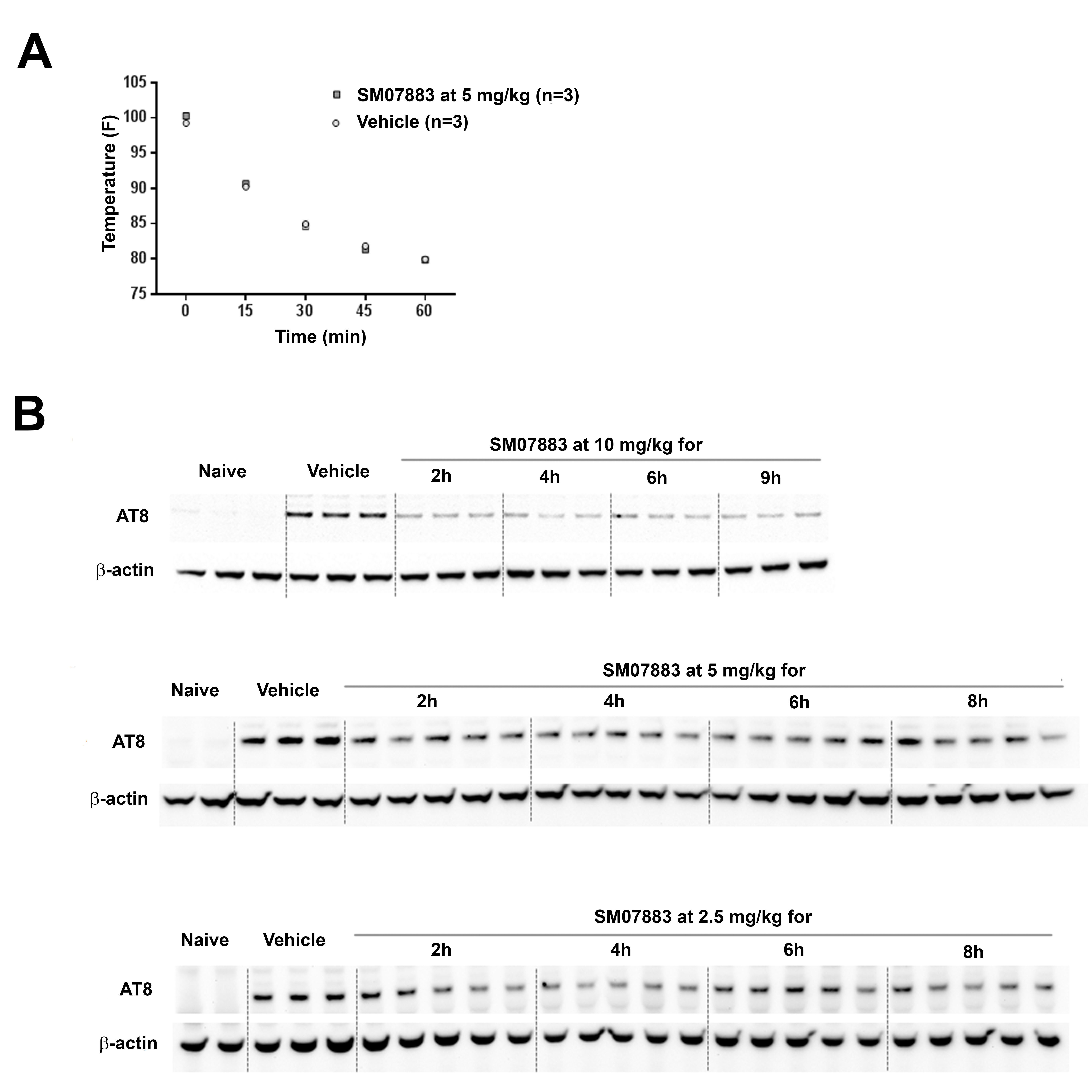

Supplement: Supplementary file 6 [file ACEL-18-e13000-s006.tif]

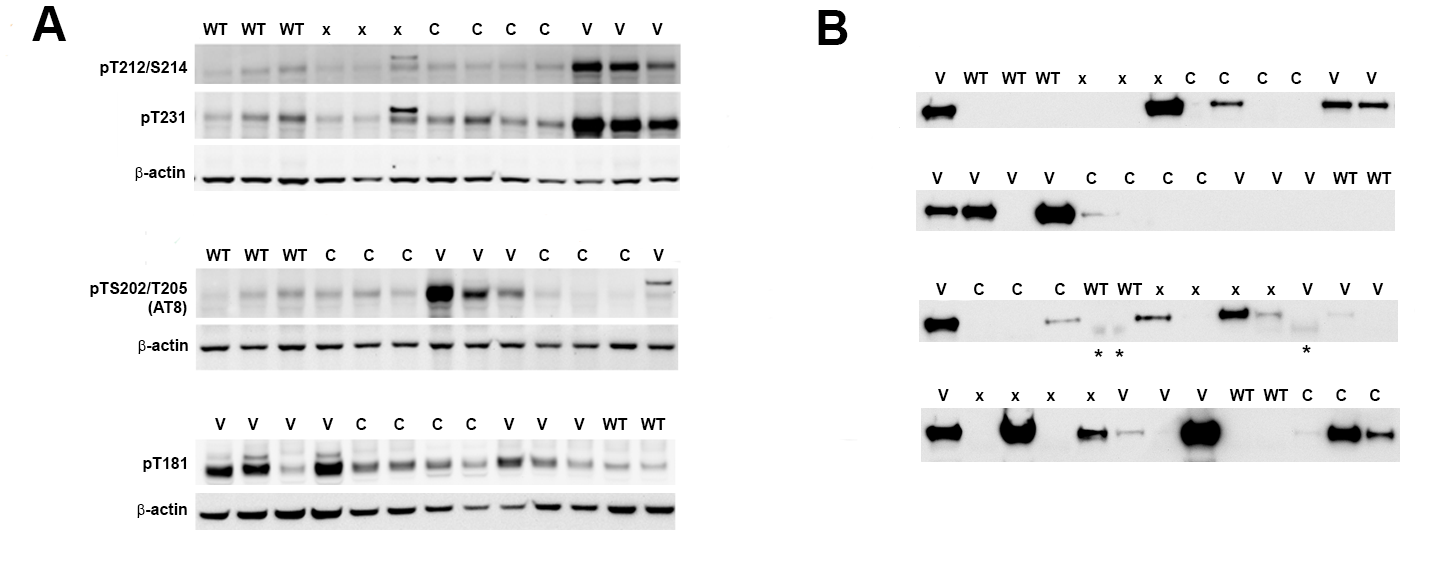

Supplement: Supplementary file 7 [file ACEL-18-e13000-s007.tif]

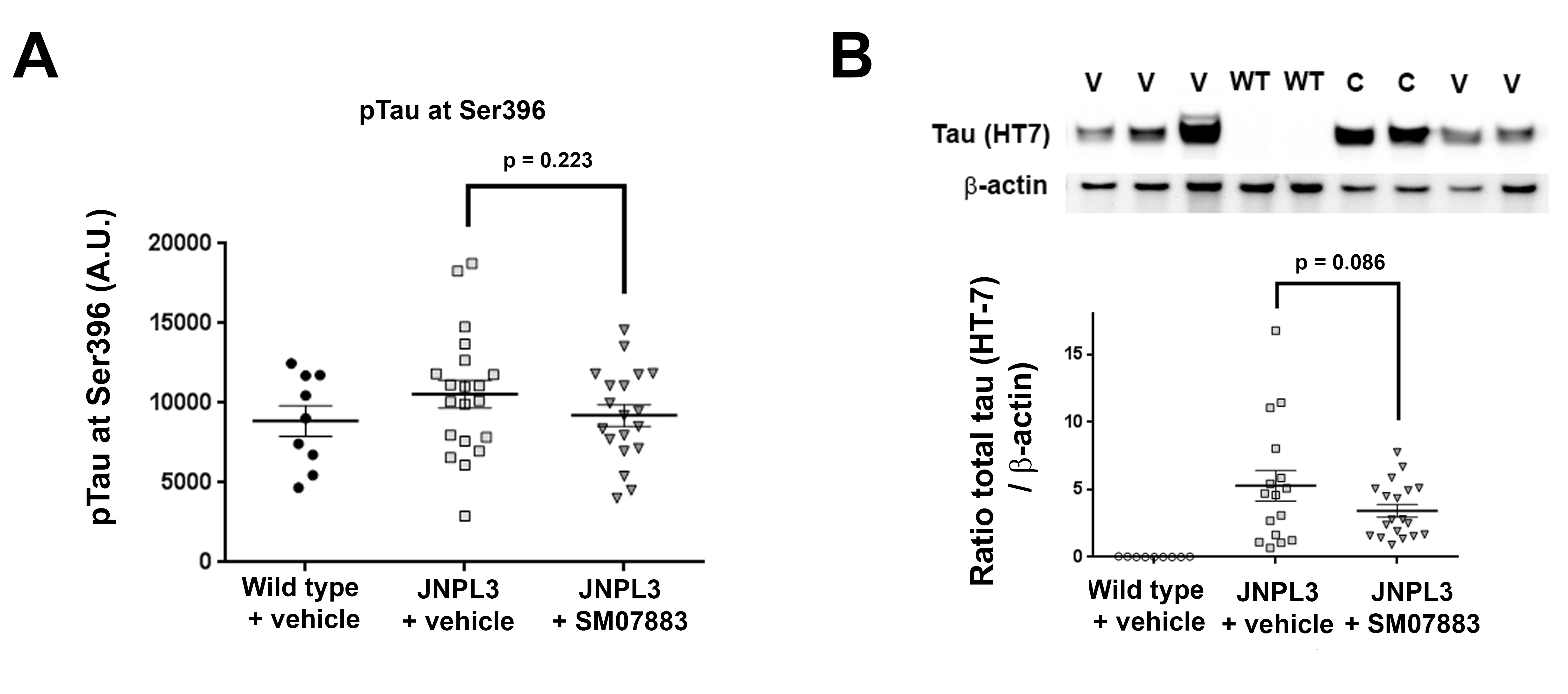

Supplement: Supplementary file 8 [file ACEL-18-e13000-s008.tif]

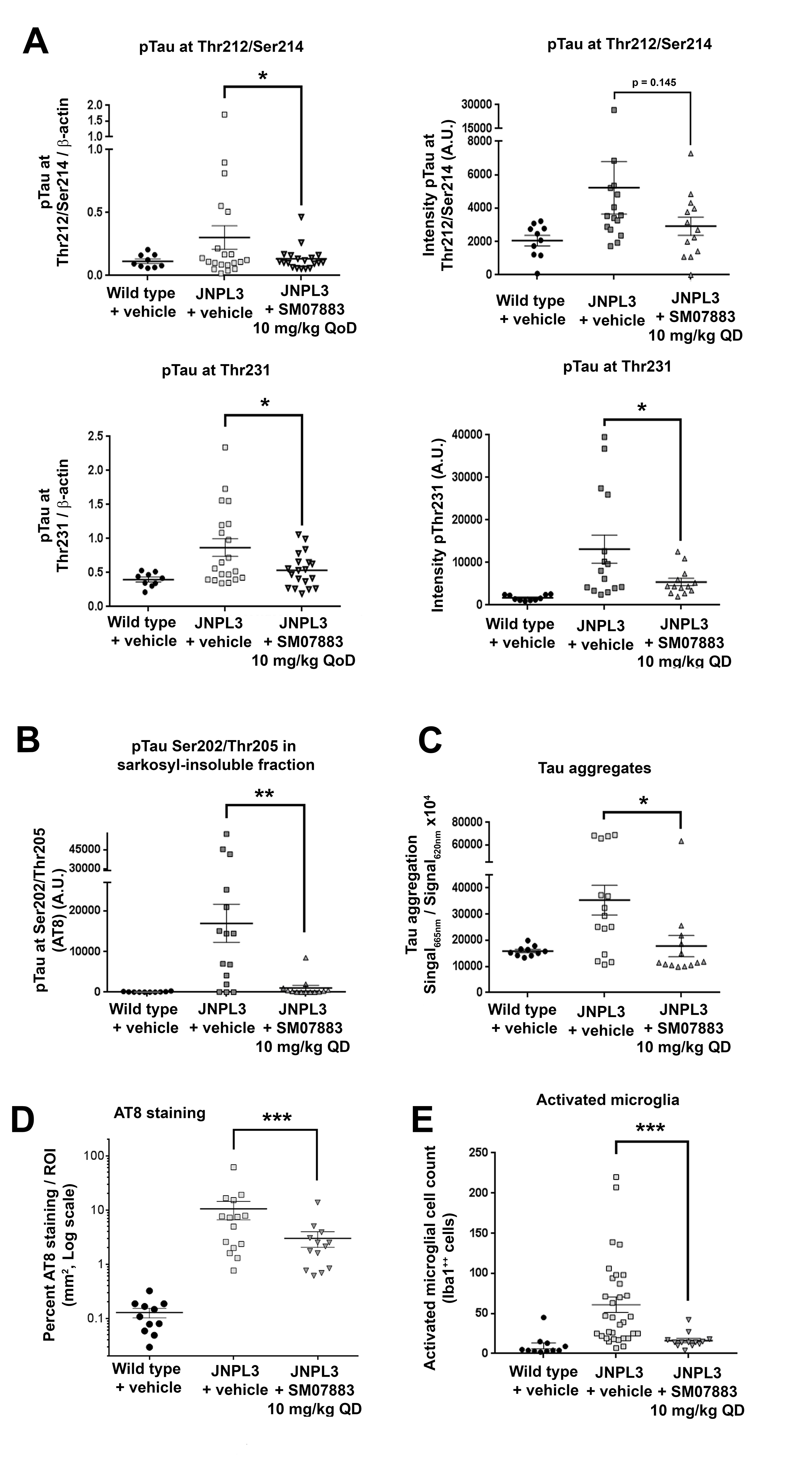

Supplement: Supplementary file 9 [file ACEL-18-e13000-s009.tif]
