# Supplementary material for: Tau pathology reduction with SM07883, a novel, potent, and selective oral DYRK1A inhibitor: A potential therapeutic for Alzheimer's disease
Source: Aging Cell. 2019 Jul 3;18(5):e13000. doi: 10.1111/acel.13000 (PMC6718548; doi:10.1111/acel.13000)
Supplement: Supplementary file 16 [file ACEL-18-e13000-s016.docx]

**Mechior et al., supplemental informatioN**

**SUPPORTING INFORMATION LISTING**

**Supplemental text file for methodology, 9 supplemental figures and 6 supplement tables and legends**

**Suppl. Fig 1:** SM07883 kinase selectivity and phosphorylated tau EC_50_ in a tau and DYRK1a overexpressing cell-based assay

**Suppl. Fig. 2.** Tau acetylation and N-glycosylation after SM07883 overnight treatment on SH-SY5Y neuroblastoma cells

**Suppl. Fig 3.** SM07883 does not increase the inclusion of MAPT exon 10

**Suppl. Fig 4.** Western blot analysis of SR proteins phosphorylation in SH-SY5Y neuroblastomas cells

**Suppl. Fig. 5.** SM07883 distribution in the Sprague Dawley rat brain

**Suppl. Fig. 6**. PK/PD relationship

**Suppl. Fig. 7.** Representative Western blots from JNPL3 brain lysates quantified in Fig. 4

**Suppl. Fig. 8.** Tau phosphorylation at Ser396 and total human tau in JNPL3 brainstem lysates

**Suppl. Fig. 9.** Reduction of tau pathology in JNPL3 mice treated with higher doses of SM07883

**Suppl. Table. 1.** Physicochemical and ADME properties of SM07883

**Suppl. Table. 2.** Correlation between kinases IC_50_ to tau phosphorylated EC_50_

**Suppl. Table 3.** Pharmacokinetic properties of SM07883

**Suppl. Table 4.** Tau pathology in JNPL3 mice versus wild type littermates

**Suppl. Table 5.** List of antibodies used for Western blot analysis and immunostaining

**Suppl. Table 6.** Statistical analyses and p values used to compare JNPL3 mice

**Supporting figures**

**
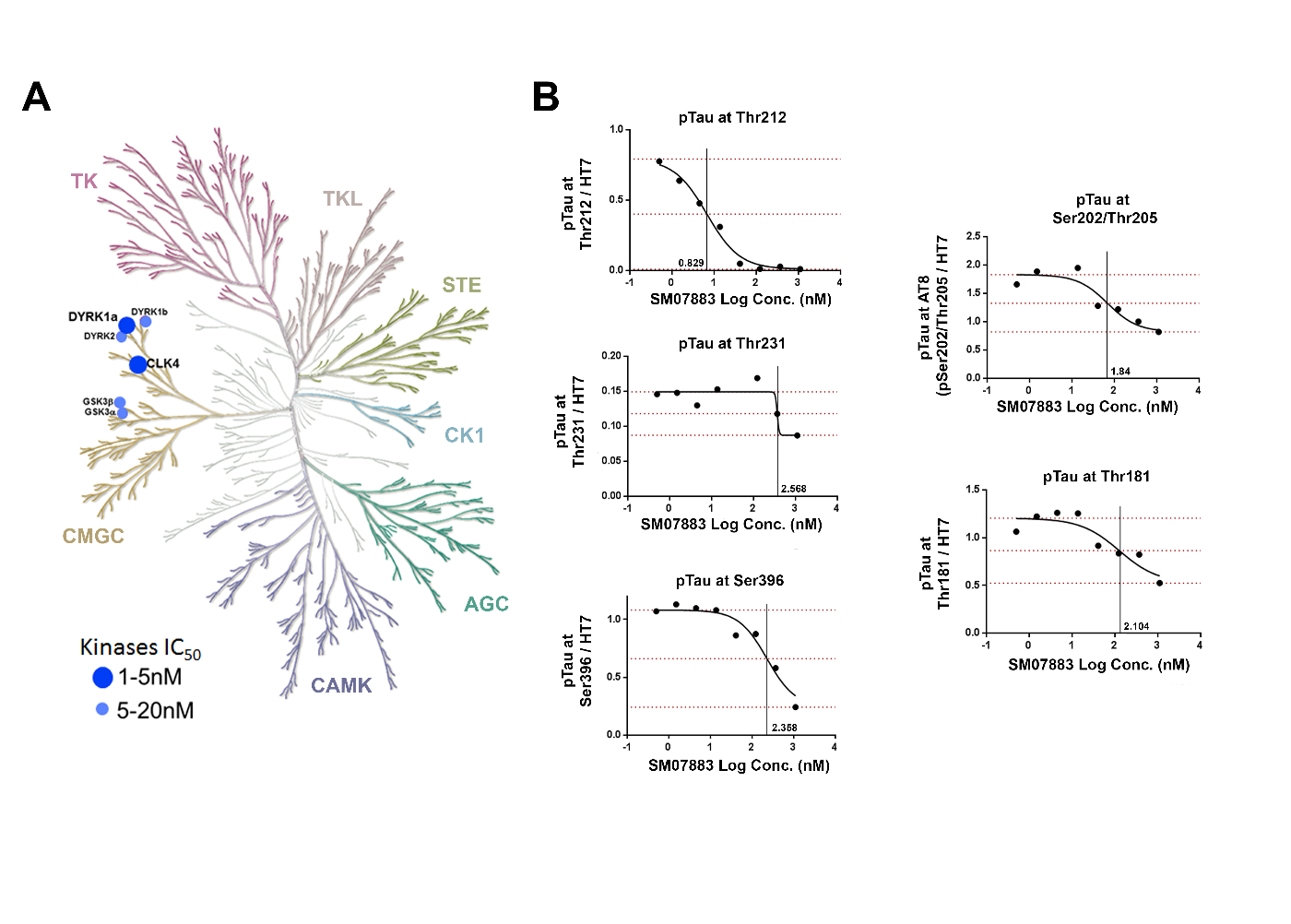
**

**Suppl. Fig 1:** SM07883 kinase selectivity and phosphorylated tau EC_50_ in a tau and DYRK1a overexpressing cell-based assay

1. Kinome render showing specificity of SM07883 kinase inhibitory activity within the CMGC kinase family. Only kinases with IC_50_ within the 15-fold range of DYRK1A are represented. Location on tree and figure were generated using KinMap (Eid et al., 2017) and the illustration a courtesy of Cell Signaling Technology, Inc. (www.cellsignal.com). **(B)** Curve representation of Western blot densitometry from the HEK293T cells assay with tau phosphorylated over total tau (HT7). Top and bottom horizontal dotted lines correspond to double MAPT/DYRK1A transfected cells (top) and maximum effect in reduction in phosphorylation (bottom) indicate the constraints of the EC_50_ curves. Horizontal dotted line in the middle indicates EC_50_. Vertical line indicates the Log concentration of SM07883 of the calculated EC_50_ value below. Inverse of Log base 10 generated the EC_50_ in nM: pSer396 = 228nM, pThr181 = 127nM. pThr212 = 7nM, pThr231 = 370nM, AT8 (pSer202/pThr205) = 69nM.


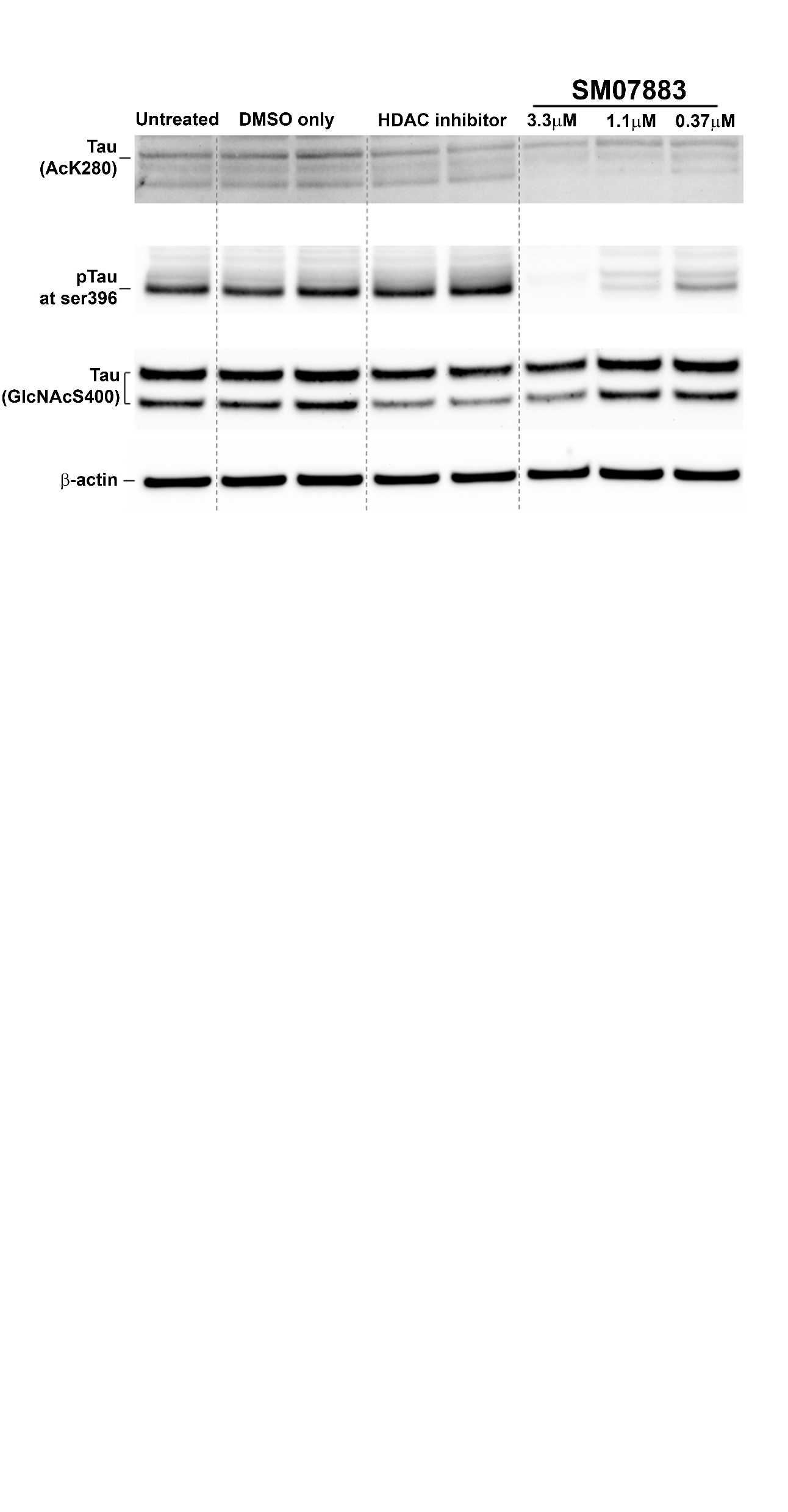


**Suppl. Fig. 2.** Tau acetylation and N-glycosylation after SM07883 overnight treatment on SH-SY5Y neuroblastoma cells

Subconfluent SH-SY5Y cells were left untreated overnight (16hrs) or treated with 2 µM of histone deacetylase inhibitor (HDAC, Catalog#113146, Cayman Chemicals, Ann Harbor, MI), SM07883 at 3.3, 1.1, 0.37 µM final in media, or DMSO only. Cell lysates were collected in RIPA and a Western blot analysis was performed with antibody specific for acetylated tau at lysine 480 (AcK280, Catalog #AS-56077, AnaSpec, Fremont, CA), phosphorylated tau at Ser396, and N-glycosylated tau at Ser400 (GlcNAcS400, Catalog #AS-55945, AnaSpec). β-actin was used as loading control.


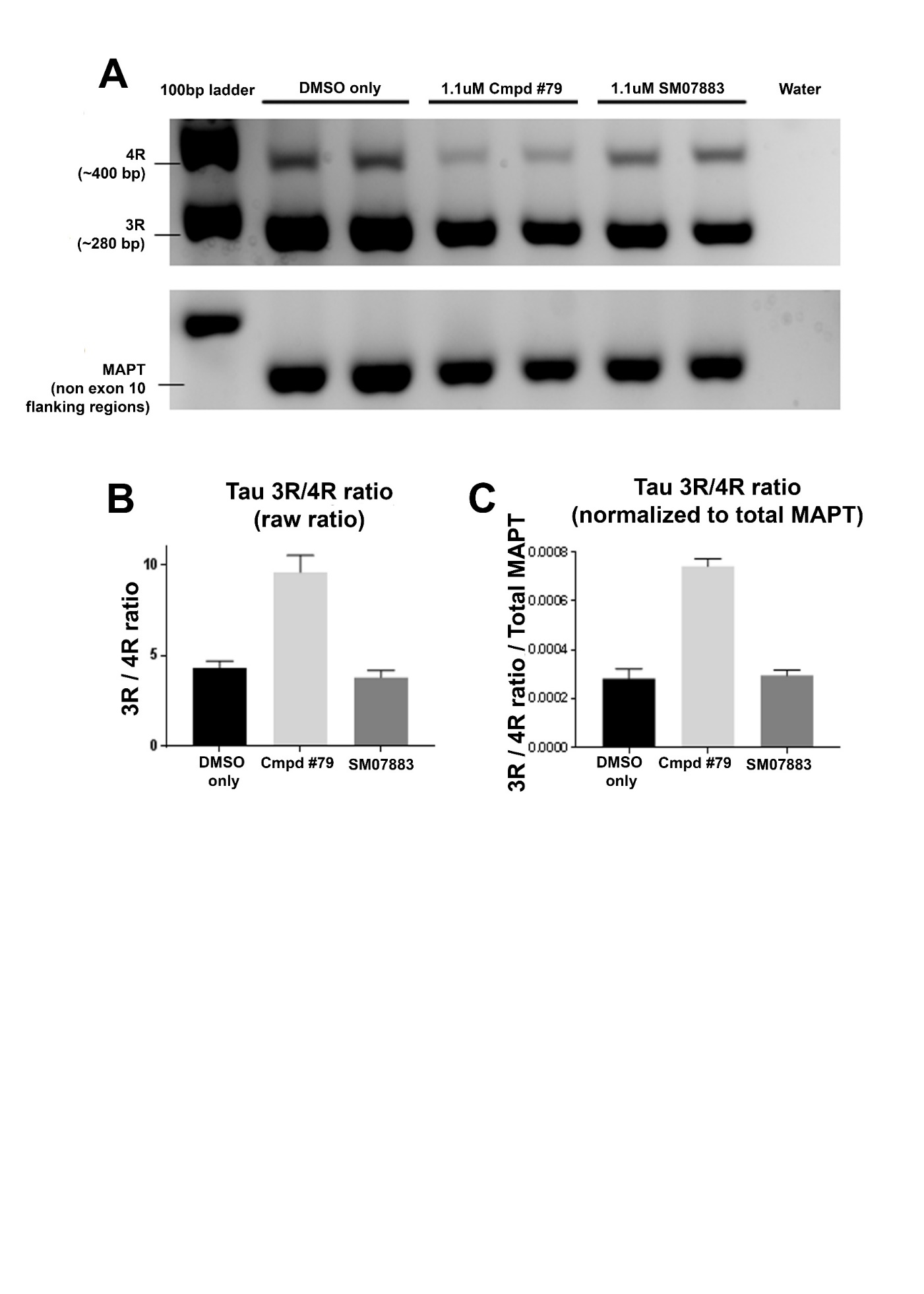


**Suppl. Fig. 3.** SM07883 does not increase the inclusion of MAPT exon 10.

**(A)** SH-SY5Y human neuroblastoma cells were treated for 16hrs with DMSO, 1.1 µM of a potent CLK inhibitor (Cmpd #79, patent US20160008365A1; CLK2 IC_50_ = 9 nM), or 1.1 µM of SM07883 and the respective cDNA was used to amplify specific regions of the MAPT gene. Reverse transcription polymerase chain reaction (RT-PCR) was used to identify splicing (3R) or inclusion of exon 10 (4R). RT-PCR cycling conditions: 98°C for 3 minutes, 30 cycles of (98°C for 10 seconds, 50°C for 30 seconds, and 68°C for 40 seconds), 68°C for 10 minutes. PCR samples were loaded onto a 2.5% agarose gel alongside a 100bp DNA ladder, and a water control. Top: RTPCR gel using the 3R/4R primer set as published by Lovino, et al. (2010). Bottom: RTPCR gel using E11/K18 primer set (Zhukareva et al*.,* 2001) amplifying a MAPT region not containing Exon 10. **(B)** The 3R/4R ratio was quantified by densitometry for each condition (left) and a ratio over total amount of tau cDNA in the samples was calculated (right). N=2 per condition.

**
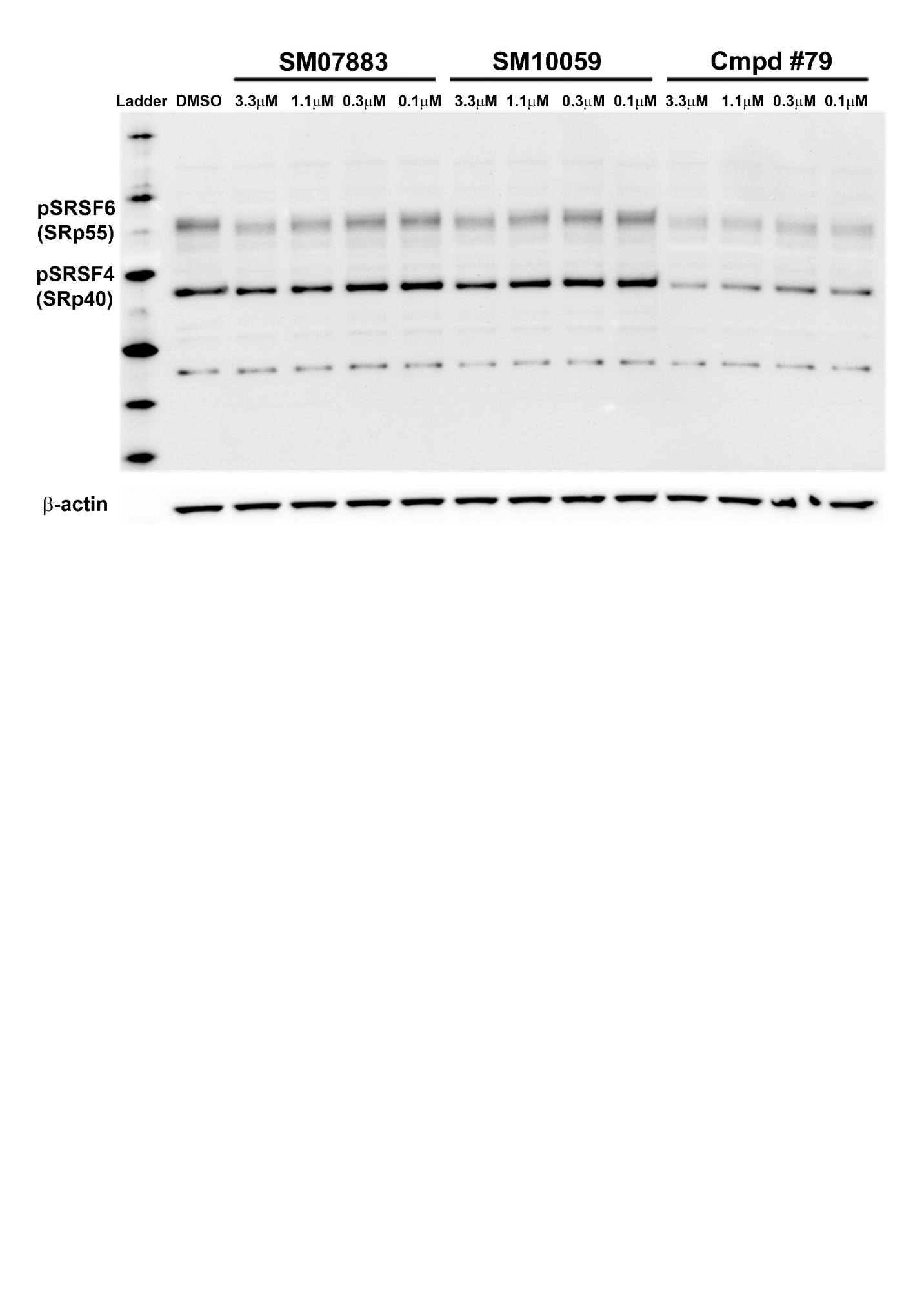
****Suppl. Fig 4.** Western blot analysis of SR proteins phosphorylation in SH-SY5Y neuroblastomas cells

SH-SY5Y human neuroblastoma cells were treated for 1hr with DMSO, or 3.3μM-0.1μM dilutions of SM07883 and SM10059, a more specific DYRK1A inhibitor (DYRK1A IC_50_ = 4nM, 5-fold selectivity over CLK4, IC_50_ = 19nM), Cmpd #79 (patent US20160008365A1), a potent CLK inhibitor as a positive control (CLK2 IC_50_ = 9nM), and cell lysates were analyzed by Western blot using a phospho-SR antibody (Catalog #MABE50, EMD Millipore, Temecula, CA). β-actin was used as loading control.


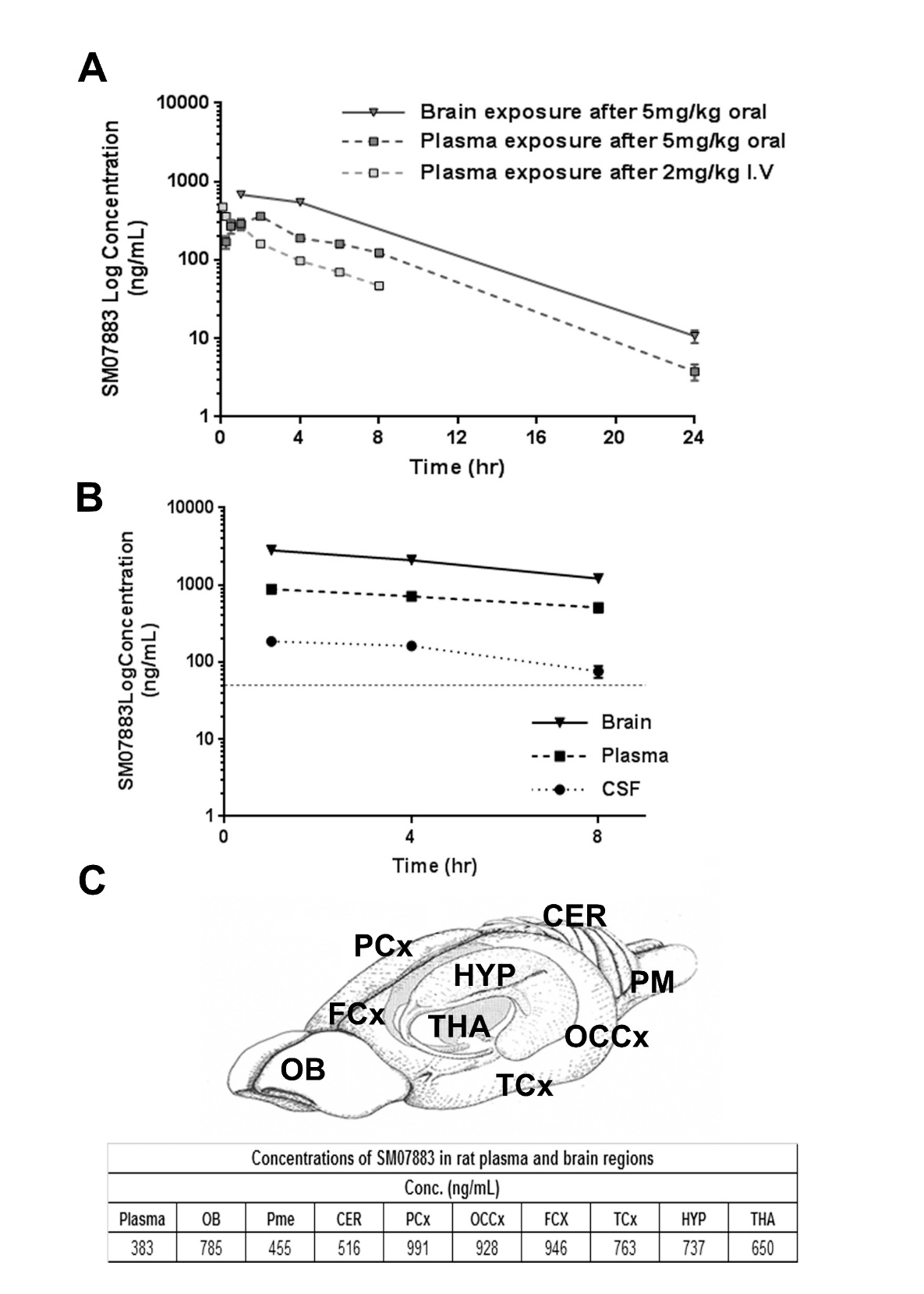


**Suppl. Fig. 5.** SM07883 distribution in the Sprague Dawley rat brain.

Brains from rats administered SM07883 (P.O., 5 mg/kg) 4hrs prior, were dissected and grossly separated in 9 regions (n=3 rats; OB: olfactory bulb, PM: pons and medulla, CER: cerebellum, PCx: parietal cortex, OCCx: occipital cortex, FCx: frontal cortex, TCx: temporal cortex, HYP: hippocampus, THA: thalamus). Plasma and brain tissue exposures are shown in the table below in ng/mL.

**
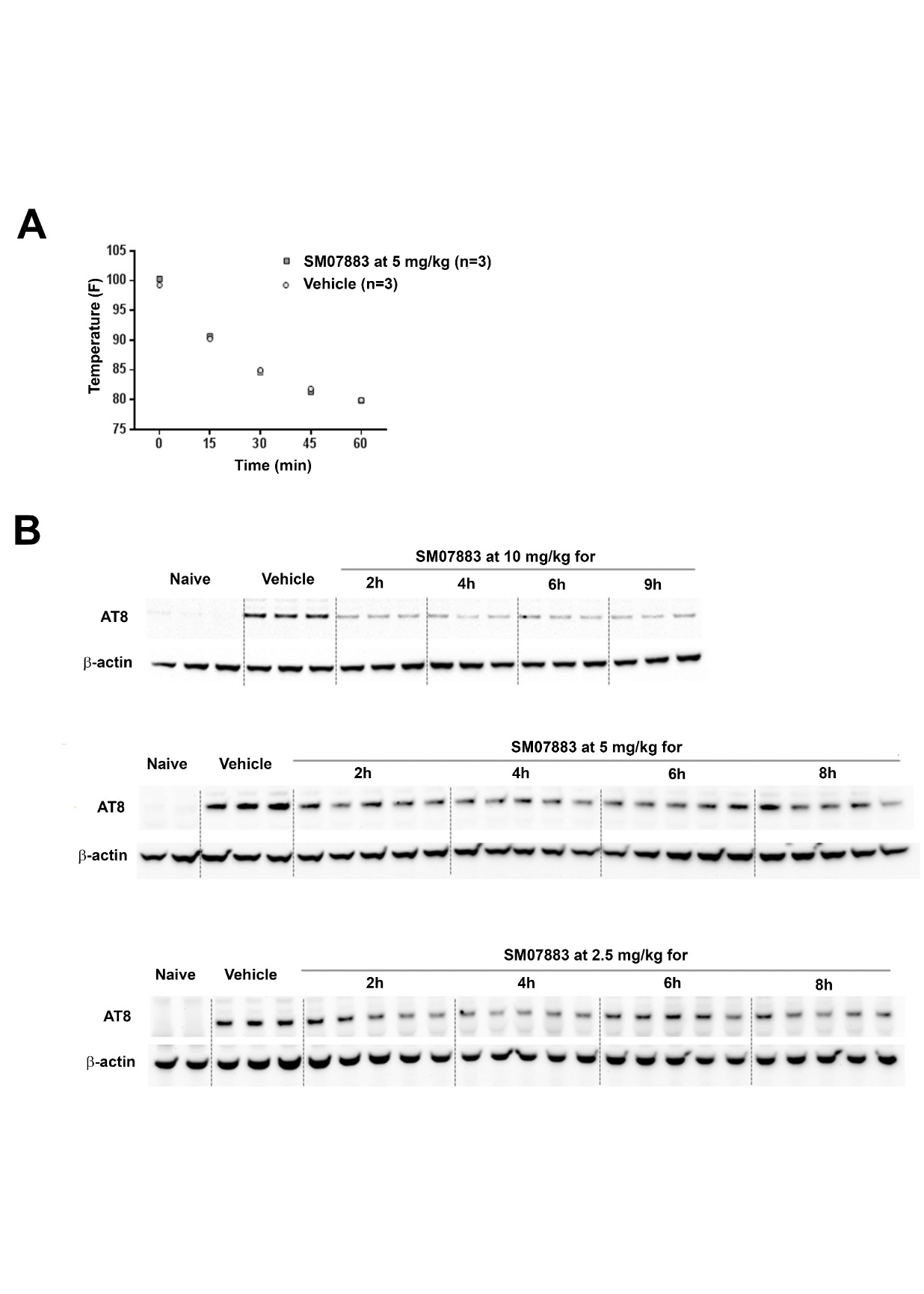
**

**Suppl. Fig. 6**. PK/PD relationship

**(A)** Administration of SM07883 did not affect anesthesia-induced decrease in body temperature. Compound (square) or vehicle (circle) were administered 3hrs prior to anesthesia by IP injection of ketamine/xylazine and body temperature was measured by rectal measurement every 15 min. n=3 animals per treatment. **(B)** Western blot supporting the time course pharmacodynamic study presented in Fig. 3E. Animals were administered a single oral dose of either 10 (top), 5 (middle), or 2.5 mg/kg (bottom) of SM07883 for 2, 4, 6, 8, or 9hrs. Anesthesia was induced 1hr prior to brain collection and lysates were analyzed by Western blotting for phosphorylated tau with the AT8 clone or β-actin.

**
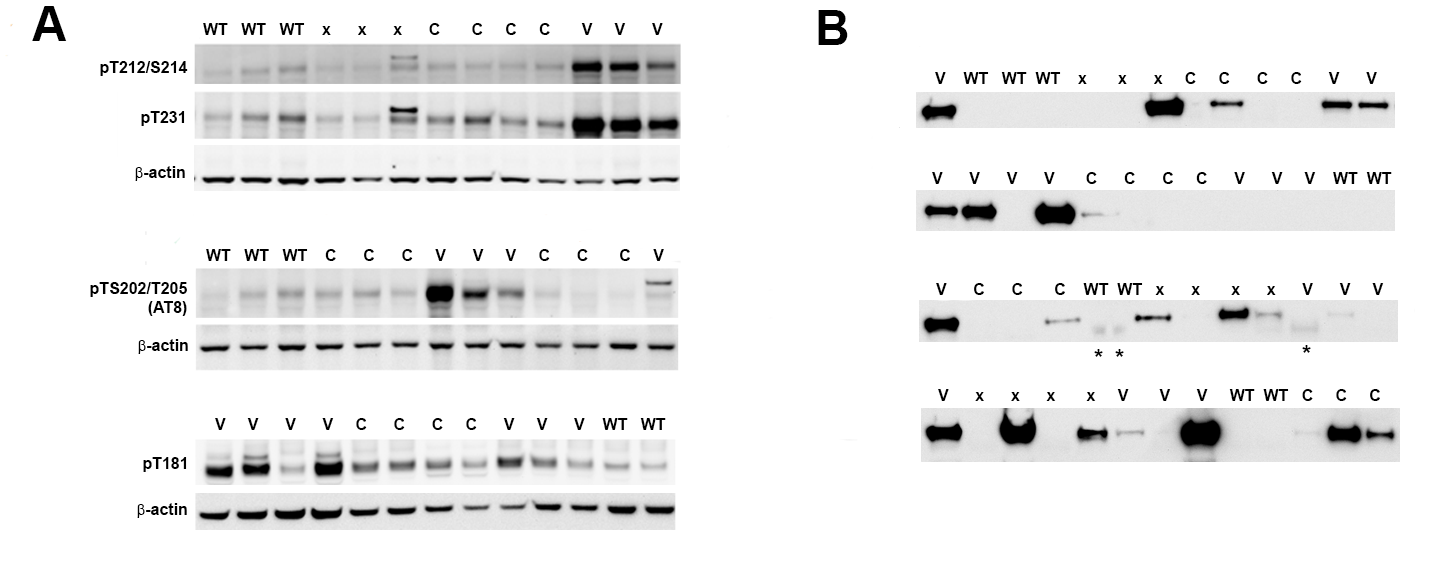
**

**Suppl. Fig. 7.** Representative Western blots from JNPL3 brain lysates quantified in Fig. 4

**(A)** Representative Western blots using JNPL3 brain lysates probed for phosphorylated tau at pT212/S214 and pT231 (top), AT8 (pS202/T205, middle) and pT181 (bottom). Densitometry was performed using NIH freeware ImageJ and data show ratio over β-actin was plotted in Prism 7 (GraphPad, La Jolla, CA) and presented in Fig. 4A. WT: wild type littermates with vehicle, V: JNPL3 administered vehicle, C: JNPL3 administered SM07883.

**(B)** Four different Western blots probed for phosphorylated tau at AT8 (pS202/T205) using sarkosyl-insoluble fraction brainstem lysates. All bands are of 64kDa or above unless indicated by an asterisk showing faint bands at the 55kDa size.

**
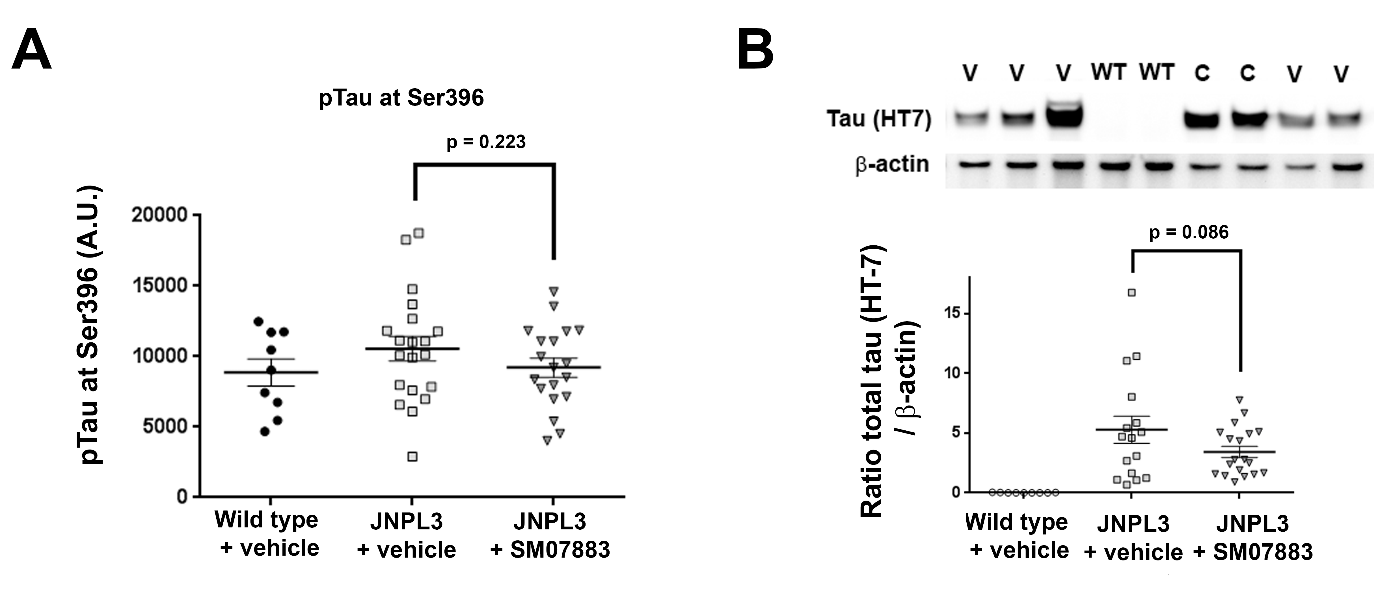
**

**Suppl. Fig. 8.** Tau phosphorylation at Ser396 and total human tau in JNPL3 brainstem lysates

**(A)** JNPL3 brainstem lysates analyzed by Western blot for tau phosphorylation at Ser396 epitopes. Vehicle-treated transgenic mice had marginally higher levels compared to wild type littermates (p=0.245) and although reduced, SM07883-treated JNPL3 mice was not significant compared to JNPL3 mice treated with vehicle (p=0.223). **(B)** Top: Representative Western blot of total human tau (Clone HT7) on brainstem lysates from JNPL3 expressing the human Tau mutation P301L. Brainstems from JNPL3 mice were collected at the termination of 3 months of treatment of a once-daily dose of vehicle (V), or SM07883 (C, 3 mg/kg) or wild type littermates administered the vehicle only (WT). Bottom: Ratio of total tau over β-actin after densitometry analysis from the complete set of lysates (Wild type, n=9, circles, JNPL3 + Vehicle, n =20, squares and JNPL3 + SM07883, n = 19, triangles; SM07883 vs JNPL3 + vehicle p=0.086).

**
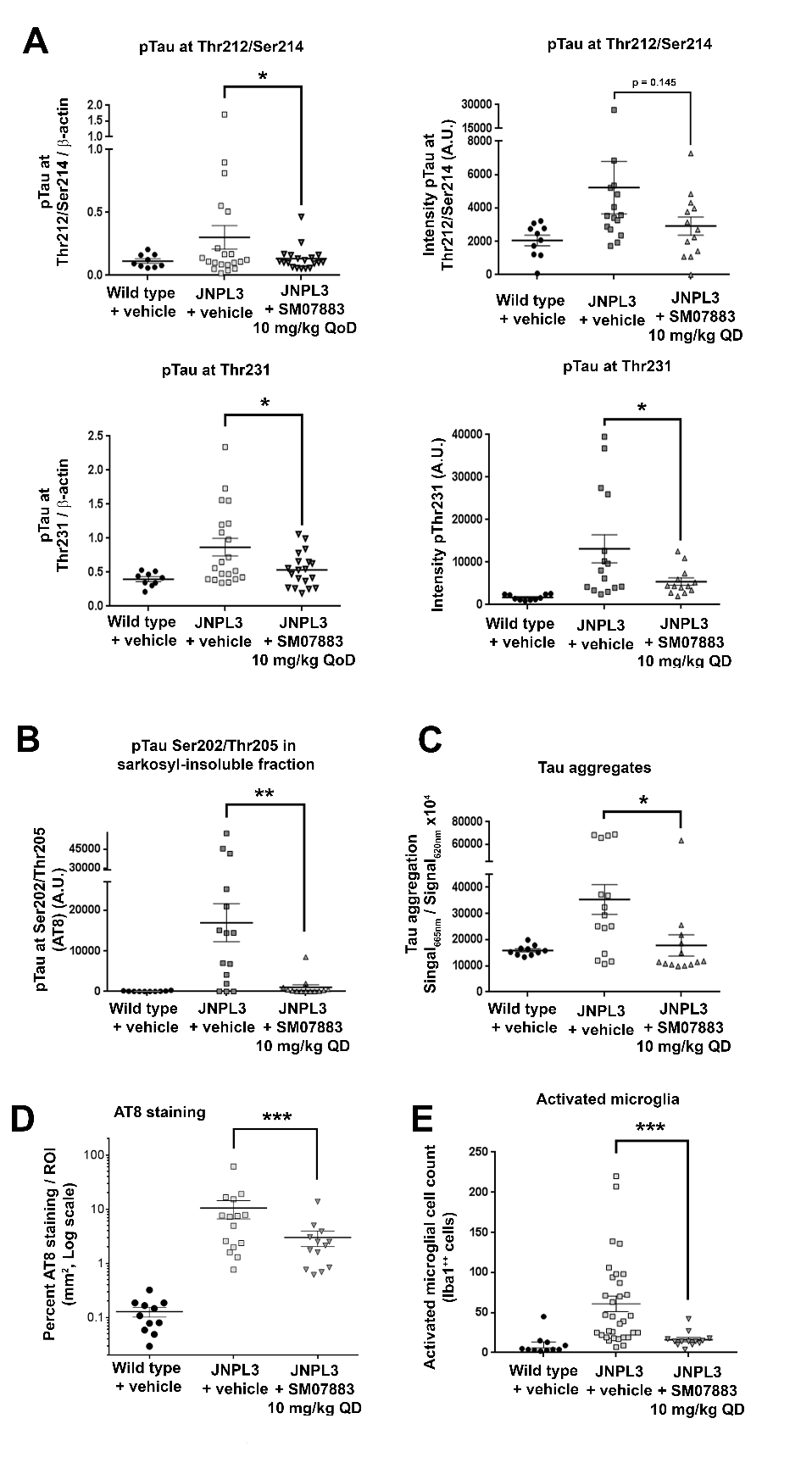
**

**Suppl. Fig. 9.** Reduction of tau pathology in JNPL3 mice treated with higher doses of SM07883
**(A)** Tau phosphorylation at Thr212/214 (Top) and Thr231 (bottom) from brainstem samples from 13-month-old JNPL3 tau mice administered SM07883 (10mg/kg, P.O., QoD [every other day] or QD [daily]). **(B)** AT8 staining from the sarkosyl-insoluble fraction (**p=0.004) and **(C)** Tau aggregates from spinal cord lysate samples from mice administered 10 mg/kg daily dose of SM07883 for 3 months or vehicle. **(D)** Total area of AT8 staining for each group in square millimeters over ROI analyzed by immunohistochemistry in the brainstem of the same animals (*** p<0.001) and **(E)** total count of activated microglia (Iba1^++^, ***p<0.001) from the same brainstems.

**Supporting tables**

**Suppl. Table. 1.** Physicochemical and ADME properties of SM07883.

Total polar surface area (tPSA) and partition coefficient between n-octanol and water (cLogP) of SM07883 were calculated by ACD/percepta 2012 software. HBD: Hydrogen Bond Donors.

**Suppl. Table. 2.** Correlation between kinases IC_50_ to tau phosphorylated EC_50_.

Comparative table of DYRK1A and GSK-3β kinase activity of SM07883 and EC_50_ from 2 cell-based assays in comparison to commercially available DYRK1A and GSK-3β small molecules inhibitor. EGCG: Epigallocatechin gallate; # Compound A is 6-Methyl-N-[3-[[3-(1-methylethoxy)propyl]carbamoyl]-1H-pyrazol-4-yl]pyridine-3-carboxamide from Uno et al. (2009); * non-ATP competitor (reported Ki from Dominguez et al. 2012).

**Suppl. Table 3.** Pharmacokinetic properties of SM07883.

Mean pharmacokinetic parameters following an intravenous (I.V.) or oral (P.O.) dose of SM07883 to male Balb/c mice, Sprague Dawley rats, Beagle dog, and Rhesus monkeys. AUC: Area under the plasma concentration versus time curve from the time of dosing extrapolated to infinity; CL: Estimated total body clearance; Vss: Estimate Volume of Distribution at steady state; %F: bioavailability. Data is mean from individual PK profile ± S.D. except ^#^ which are mean concentrations from multiple animals. NA: Not available.

| **Outcome** | **Wild Type** | **JNPL3 + Vehicle** | **P-value** | **Figure** |
| --- | --- | --- | --- | --- |
| **pTau at Thr212/Ser214** | 0.115 (0.053, 0.250) | 0.303 (0.180, 0.510) | 0.129 | 4A |
| **pTau at Thr231** | 0.399 (0.275, 0.579) | 0.869 (0.677, 1.114) | **0.006** | 4A |
| **pTau at Ser202/Thr205** | 0.586 (0.300, 1.14) | 0.959 (0.605, 1.519) | 0.294 | 4A |
| **pTau at Thr181** | 0.196 (0.136, 0.283) | 0.961 (0.752, 1.228) | **<0.001** | 4A |
| **Sarkosyl-insoluble (AT8)** | 16 [11, 45] | 1438.5 [95, 10542.5] | **<0.001*** | 4B |
| **Tau Aggregation by HTRF** | 15325.2 (9791.0, 23987.4) | 25296.2 (18583.9, 34432.84) | 0.111 | 4C |
| **Tau inclusions (immunostaining)** | 0.128 (0.050, 0.331) | 7.666 (3.655, 16.078) | **0.042** | 5E |
| **GFAP (ELISA)** | 399.098 (276.113, 576.861) | 820.062 (631.997, 1064.089) | **0.010** | 5F |
| **Iba1++ cell count** | 9.36 (7.72, 11.36) | 60.8 (58.2, 63.6) | **<0.001†** | 5G |
| **pTau at Ser396** | 8874.56 (7018.60, 11221.30) | 10562.5 (9024.29, 12362.93) | 0.245 | Suppl. 8A |
| **Total human Tau** | 0.02 (0.01, 0.03) | 5.28 (3.28, 8.50) | **0.002** | Suppl. 8B |

**Suppl. Table 4.** Tau pathology in JNPL3 mice versus wild type littermates

Statistical analyses show significant degree of pathology in JNPL3 mice treated with vehicle (PO, QD) for 3 months compared to age matched wild type littermates treated with vehicle (PO, QD). Study was not powered to characterize JNPL3 (n=19) vs wild type (n=9) mice. *Median [Interquartile Range (25th, 75th quartile)] and Wilcoxon-Mann-Whitney test reported. †Mean (95% CI) and Poisson generalized linear model reported. Otherwise, mean (95% CI) and Gamma generalized linear model reported.

| **SM07883 3 mg/kg QD** | | | | |
| --- | --- | --- | --- | --- |
| **Outcome** | **JNPL3 + SM07883** | **JNPL3 + Vehicle** | **P-value** | **Figure** |
| **pTau at Thr212/Ser214** | 0.106 (0.063, 0.181) | 0.303 (0.181, 0.507) | **0.023** | 4A |
| **pTau at Thr231** | 0.423 (0.329, 0.543) | 0.869 (0.680, 1.109) | **<0.001** | 4A |
| **pTau at Ser202/Thr205** | 0.421 (0.276, 0.641) | 0.959 (0.630, 1.460) | **0.017** | 4A |
| **pTau at Thr181** | 0.658 (0.498, 0.869) | 0.961 (0.733, 1.260) | 0.064 | 4A |
| **Sarkosyl-insoluble (AT8)** | 61 [25, 477] | 1438.5 [95, 10542.5] | **0.010*** | 4B |
| **Tau Aggregation by HTRF** | 16670.0 (12362.7, 22478.1) | 25297.3 (18760.8, 34111.2) | 0.062 | 4C |
| **Tau inclusions (immunostaining)** | 1.582 (0.756, 3.309) | 7.666 (3.592, 16.361) | **0.039** | 5E |
| **GFAP (ELISA)** | 377.750 (297.981, 478.875) | 820.062 (642.698, 1,046.373) | **<0.001** | 5F |
| **Iba1++ cell count** | 52.1 (49.0, 55.5) | 60.8 (58.2, 63.6) | **<0.001†** | 5G |
| **pTau at Ser396** | 9219.8 (7891.6, 10771.6) | 10562.5 (9076.5, 12291.8) | 0.223 | Suppl. 8A |
| **Total human Tau** | 3.43 (2.49, 4.76) | 5.28 (3.70, 7.54) | 0.086 | Suppl. 8B |
|  |  |  |  |  |
| **SM07883 10 mg/kg QD** | | | | |
| **Outcome** | **JNPL3 + SM07883** | **JNPL3 + Vehicle** | **P-value** | **Figure** |
| **pTau at Thr212/Ser214** | 2911.85 (1719.00, 4932.42) | 5219.20 (3195.34, 8524.93) | 0.145 | Suppl. 9A |
| **pTau at Thr231** | 5425.08 (3488.78, 8436.04) | 13139.47 (8744.48, 19893.92) | **0.015** | Suppl. 9A |
| **Sarkosyl-insoluble (AT8)** | 195 [8, 779] | 14380 [1929, 25207] | **0.004*** | Suppl. 9B |
| **Tau Aggregation by HTRF** | 17763.4 (12003.6, 26286.8) | 35323.2 (24524.5, 50876.7) | **0.024** | Suppl. 9C |
| **Tau inclusions (immunostaining)** | 2.06 [0.81, 2.94] | 10.25 [4.78, 21.75] | **<0.001** | Suppl. 9D |
| **Iba1++ cell count** | 16.2 (14.2, 18.6) | 60.8 (58.2, 63.6) | **<0.001†** | Suppl. 9E |
|  |  |  |  |  |
| **SM07883 10 mg/kg QoD** | | | | |
| **Outcome** | **JNPL3 + SM07883** | **JNPL3 + Vehicle** | **P-value** | **Figure** |
| **pTau at Thr212/Ser214** | 0.133 (0.081, 0.220) | 0.303 (0.186, 0.493) | **0.044** | Suppl. 9A |
| **pTau at Thr231** | 0.536 (0.414, 0.694) | 0.869 (0.675, 1.117) | **0.013** | Suppl. 9A |

**Suppl. Table 5.** Statistical description and p values used to compare JNPL3 mice treated with vehicle vs SM07883 3 or 10 mg/kg QD or 10 mg/kg QoD. *Median [Interquartile Range (25th, 75th quartile)] and Wilcoxon-Mann-Whitney test reported. †Mean (95% CI) and Poisson generalized linear model reported. Otherwise, mean (95% CI) and Gamma generalized linear model reported.

| **Target Name** | **Host Species** | **Company** | **Catalog** | **Dilution** |
| --- | --- | --- | --- | --- |
| DYRK1A | Rabbit | CST | 8765S | 1:500 |
| pTau (S396) [EPR2731] | Rabbit | abcam | ab109390 | 1:1000 |
| pTau (T231) | Mouse | Thermo Fisher | MN1040 | 1:500 |
| pTau (T212) | Rabbit | Thermo Fisher | 44-740G | 1:500 |
| pTau (AT8, S202/T205) | Mouse | Thermo Fisher | MN1020 | 1:500 |
| Tau (HT7) | Mouse | Thermo Fisher | MN1000 | 1:1000 |
| pTau (T181) | Mouse | Thermo Fisher | MN1050 | 1:500 |
| pTau (T212/S214) | Mouse | Thermo Fisher | MN1060 | 1:500 |
| GAPDH (HRP conjugate) | Rabbit | CST | 8884S | 1:2000 |
| β-actin (HRP conjugate) | Rabbit | CST | 5125S | 1:2000 |

**Suppl. Table 6.** List of antibodies used for Western blot analyses

**Supplemental methods:**

Cell Transfection and ELISA assays for pTau at Thr212 and Ser396

### For the generation of the DYRK1A- and MAPT-overexpressing cells, HEK293T cells were transiently transfected with DYRK1A and MAPT genes. Specifically, expression vectors pCMV6-XL4 and pCMV6-Entry expressing DYRK1A and MAPT, alongside an empty vector control (pCI, OriGene, Catalog #SC314641 and RC216166, respectively; Promega, Catalog #E1731) and the material was amplified and purified using a custom MaxiPrep service (GENEWIZ, South Plainfield, NJ). HEK293T cells were transfected with Lipofectamine™ 3000 Transfection Reagent (Thermo Fisher) diluted in Opti-MEM medium according to the manufacturer’s suggested protocol.

For ELISA assays, native SH-SY5Y cells, or transiently double-transfected HEK293T cells were dissociated by treatment with trypsin-EDTA and seeded in 96-well flat-bottom cell culturing plate before administration of 3-fold serial dilution of SM07883 overnight.

The pSer396Tau sandwich ELISA was performed with a pS396Tau Human ELISA kit (Invitrogen), and was carried out by diluting 80µL of SH-SY5Y lysate supernatant into 20µL of sample diluent and completing the assay according to the manufacturer’s suggested protocol.

Following compound treatment, cells were washed and lysed with 1x RIPA buffer containing phosphatase and protease inhibitors transferred to 96-well V-bottom collection plates for centrifugation and removal of insoluble material.

The pTau Thr212 ELISA 96-well flat-bottom plates, were coated with anti-HT7 capture antibody (Catalog MN1000, Thermo Fisher, Waltham, CA) diluted 1:300 in 1X PBS at 4°C. After overnight incubation, coated plates were washed with 1× PBS containing 0.05% Tween-20 and blocked for 1hr 2% BSA (A7030-50G, Sigma-Aldrich, St. Louis, MO), then washed prior to sample loading.

For the pTau Thr212 sandwich ELISA, cell lysates were directly transferred to the anti-HT7 capture, antibody-coated, and BSA-blocked ELISA plates for 2hrs. Supernatant was then removed, and plates were washed with 1× PBS-0.05% Tween-20, followed by addition of 100 µL per well anti-phosphorylated tau Thr212 antibody (Catalog 44-740G, Thermo Fisher) diluted 1:200 in 1× PBS for 2hrs. Plates were washed and probed with an anti-rabbit/HRP conjugate (Cell Signaling Technology, 7074S) for 1hr then washed before a TMB substrate solution (Catalog N301, Thermo Fisher) was added. When a deep blue color was observed, 100 µL per well of stop solution (Catalog N600, Thermo Fisher) was added.

Colorimetric detection of pT212Tau or pS396Tau signal was read at 450 nm using the Cytation 3 Cell Imaging Multi-Mode Reader (BioTek, Winooski, VT). The signal was used to plot, draw the curve fitting, and determine the EC_50_ value for SM07883 in Prism 7 (GraphPad, La Jolla, CA). EC_50_ were averaged from n=4 and n=6 individual experiments for the Thr212 and Ser 396 assays respectively.

The transient transfection of HEK293T cells with DYRK1A and MAPT expression vectors (catalog numbers above) was confirmed with Western blot analysis of related targets with primary antibodies listed in [Suppl. Table 4](#Suptab4).

**Additional animal procedures and ethics approval**

This study was carried out in strict accordance with the recommendations in the Guide for the Care and Use of Laboratory Animals of the National Institutes of Health and approved by the Committee at Samumed, LLC. All experiments were approved by the Institutional Animal Care and Use Committee. Animals were housed 2–5 per cage, kept on 12hr light/dark cycle and were given *ad libitum* access to food and water. Tail biopsy genotyping was performed to ensure that JNPL3 transgenic mice were homozygous for the human tau gene and not homozygous for the Pde6b^rd1^ mutation.

Plasma and tissue processing for bioanalytical studies

Following a single Intravenous (I.V.) bolus or oral (P.O.) dose to Male Balb/c mice, approximately 0.1 mL whole blood was collected via the cheek vein (submandibular) according to an alternate bleeding schedule (n=3/timepoint/route) at 0.083 (I.V.), 0.25, 0.5, 1, 2, 4, 6, 8, and 24hrs post dose into tubes containing K_2_EDTA anticoagulant and plasma was harvested by centrifugation. CSF and brain tissue were collected at 1, 4, 8, and 24hrs post dose. A bioanalytical method was developed and used to analyze SM07883 concentrations in mouse plasma, brain, and CSF. The method was both sensitive and selective, and employed high-performance liquid chromatography (HPLC) coupled with tandem mass spectrometric (MS/MS) detection.

Plasma and brain homogenate samples were analyzed by LC/MS/MS in ESI (+) MRM mode. Analyses were performed on an Applied Biosystems API4000 Q-Trap with Shimadzu 20 Prominence HPLC and autosampler. The chromatograms of the plasma and brain homogenate samples were integrated and calibrated using Analyst 1.6.2 (AB Sciex, Framingham, MA). Linear regression with 1/x^2^ weighting and internal standardization was used for standard calibration, with an acceptance criterion of ±30% of nominal standard concentration.

Scoring of grip and time to fall in the wire-hang model

Assessment was based on a composite score using a 1-5 scoring system for falling and 0-5 scoring system for grip as described in Morgan et al., 2008 and Garcia et al., 2003. The maximum time for each trial was 60 seconds and the trials were repeated 3 times at 1hr interval 3 weeks prior to the first treatment administration and after 14 weeks of treatment.

Agility and grip rating system: (0) corresponds to when the animal is unable to hang onto the string for any length of time; “1” if the animal hangs by its forepaws for 60 seconds, (2) if the animal makes an attempt to pull itself up onto the string, (3) if the mouse places both forepaws and at least one hind paw on the string, (4) if the animal places all four paws and tail around the string with some lateral movement, (5) if the animal escapes to the support platform within the 60sec period.

Time to fall rating system: (1) if the animal falls ≤ 5 sec, (2) if the animal falls >5 sec and ≤10 sec, (3) if the animal falls >10 sec and ≤20 sec, (4) when the animal falls >20 sec and ≤30 sec, and (5) if the animal remains on the wire >30 sec or reaches the platform at any time.

**Clinical score in JNPL3**

Clinical assessment for the presence of motor and behavioral dysfunction at termination of the study in 13-month old wild type and JNPL3 mice treated PO daily with vehicle or SM07883 are summarized in Table 2. Mice were monitored for gross clinical score and clinical signs typical of the JNPL3 transgene-induced disease phenotype such as tremors, limited mobility and reduction in grooming. Symptoms were categorized into three stages. Animals in the pre-symptomatic stage exhibited normal spontaneous activity level, posture, and gait. Progression of disease to early symptomatic stage included moderate hunched posture and mild resting state tremor. The fully symptomatic stage was characterized by pronounced hunched back and severe tremors. Analysis was performed by an operator blinded from treatment.

Immunostaining of mouse brains

Brain hemispheres were treated overnight in 20% glycerol and 2% DMSO, to prevent freeze-artifacts, then embedded in a proprietary gelatin matrix before blocks of multiple brains were immersed in cold isopentane. 30 µm sections were collected for staining after hydrogen peroxide treatment, TritonX-100 permeabilization, and blocking steps. A similar biotinylated clone of AT8 was used as primary antibody (Thermo Fisher). A biotinylated secondary anti-mouse IgG antibody (Thermo Fisher) was applied, and the signal was amplified with the ABC solution (avidin-biotin-HRP complex; Vectastain Elite ABC, Vector, Burlingame, CA). After rinsing, the sections were treated with diaminobenzidine tetrahydrochloride (DAB) and hydrogen peroxide to create a visible reaction product. Quantitative densitometric and particle analysis of the images were captured using an LE120 slide scanning system from Huron Digital Pathology. Each image was captured at 10x resolution (1 μm/pixel). Quantification for AT8 was performed blindly. The brains from the 2 early deaths in the vehicle group were not preserved in similar conditions as the rest of the animals and brain tissues were not found suitable for immunohistological staining. The same group of wild type animals are presented in both Suppl. Fig. 8 and Fig. 5E.

**GFAP measurement in JNPL3 spinal cord lysates**

The SimpleStep ELISA (Cat# AB233621, Abcam, Cambridge, UK) employed an affinity tag labeled capture antibody and a reporter conjugated detector antibody which immunocapture the sample analyte in spinal cord RIPA lysates. This entire complex (capture antibody/analyte/detector antibody) was in turn immobilized via immunoaffinity of an anti-tag antibody coating the well. Lysates samples had to be further diluted 1 to 80,000-fold in 1X cell extraction buffer PTR provided by the manufacturer for measurement to fit within the working range of the assay.

**Supporting references**

Domínguez, J. M., Fuertes, A., Orozco, L., del Monte-Millán, M., Delgado, E., & Medina, M. (2012). Evidence for irreversible inhibition of glycogen synthase kinase-3β by tideglusib. *The Journal of Biological Chemistry*, *287*(2), 893–904. <https://doi.org/10.1074/jbc.M111.306472>

Eid, S., Turk, S., Volkamer, A., Rippmann, F., & Fulle, S. (2017). KinMap: a web-based tool for interactive navigation through human kinome data. *BMC Bioinformatics*, *18*(1), 16. <https://doi.org/10.1186/s12859-016-1433-7>

Garcia, M.F. (2003). An assessment of cognitive and sensorimotor deficits associated with appsw and p301l mouse models of alzheimer's disease. *Scholar Commons, University of Florida*, 6-1-2003.

Morgan, D., Munireddy, S., Alamed, J., DeLeon, J., Diamond, D.M., Bickford, P., Hutton, M., …Gordon, M.N. (2008). Apparent Behavioral Benefits of Tau Overexpression in P301L Tau Transgenic Mice*. J. Alzheimers Dis., 15*(4), 605-14.

Lovino, M., Patani, R., Watts, C., Chandran, S., & Spillantini, M. G. (2010). Human Stem Cell-Derived Neurons: A System to Study Human Tau Function and Dysfunction. *PLoS ONE*, *5*(11), e13947. <https://doi.org/10.1371/journal.pone.0013947>

Uno, Y., Iwashita, H., Tsukamoto, T., Uchiyama, N., Kawamoto, T., Kori, M., & Nakanishi, A. (2009). Efficacy of a novel, orally active GSK-3 inhibitor 6-Methyl-N-[3-[[3-(1-methylethoxy)propyl]carbamoyl]-1H-pyrazol-4-yl]pyridine-3-carboxamide in tau transgenic mice. *Brain Research*, *1296*, 148–163. <https://doi.org/10.1016/j.brainres.2009.08.034>

Zhukareva, V., Vogelsberg-Ragaglia, V., Van Deerlin, V. M., Bruce, J., Shuck, T., Grossman, M., … Lee, V. M. (2001). Loss of brain tau defines novel sporadic and familial tauopathies with frontotemporal dementia. *Annals of Neurology*, *49*(2), 165–175. Retrieved from <http://www.ncbi.nlm.nih.gov/pubmed/11220736>
